# Global distribution of zoonotic digenetic trematodes: a scoping review

**DOI:** 10.1186/s40249-024-01208-1

**Published:** 2024-06-14

**Authors:** Yue Hu, Rong-Jian Zhan, Shi-Lin Lu, Yi-Yang Zhang, Min-Yu Zhou, Hui Huang, Ding-Ding Wang, Tao Zhang, Zi-Xin Huang, Yun-Fei Zhou, Zhi-Yue Lv

**Affiliations:** 1 Key Laboratory of Tropical Disease Control (Sun Yat-Sen University), Ministry of Education, Guangzhou, Guangdong China; 2 Provincial Engineering Technology Research Center for Biological Vector Control, Guangzhou, Guangdong China; 3 Department of Otorhinolaryngology, The First Affiliated Hospital of Sun Yat-Sen University, Guangzhou, Guangdong China; 4 NHC Key Laboratory of Tropical Disease Control, Hainan Medical University, Haikou, Hainan China; 5 Hainan General Hospital, Hainan Affiliated Hospital of Hainan Medical University, Haikou, Hainan China

**Keywords:** Digenetic trematode, Epidemiology, Spatio-temporal distribution

## Abstract

**Background:**

Digenetic trematodes, including blood flukes, intestinal flukes, liver flukes, lung flukes, and pancreatic flukes, are highly diverse and distributed widely. They affect at least 200 million people worldwide, so better understanding of their global distribution and prevalence are crucial for controlling and preventing human trematodiosis. Hence, this scoping review aims to conduct a comprehensive investigation on the spatio-temporal distribution and epidemiology of some important zoonotic digenetic trematodes.

**Methods:**

We conducted a scoping review by searching PubMed, Web of Science, Google Scholar, China National Knowledge Infrastructure, and Wanfang databases for articles, reviews, and case reports of zoonotic digenetic trematodes, without any restrictions on the year of publication. We followed the inclusion and exclusion criteria to identify relevant studies. And relevant information of the identified studies were collected and summarized.

**Results:**

We identified a total of 470 articles that met the inclusion criteria and were included in the review finally. Our analysis revealed the prevalence and global distribution of species in *Schistosoma*, *Echinostoma*, *Isthmiophora*, *Echinochasmus*, *Paragonimus*, Opisthorchiidae, Fasciolidae, Heterophyidae, and *Eurytrema*. Although some flukes are distributed worldwide, developing countries in Asia and Africa are still the most prevalent areas. Furthermore, there were some overlaps between the distribution of zoonotic digenetic trematodes from the same genus, and the prevalence of some zoonotic digenetic trematodes was not entirely consistent with their global distribution. The temporal disparities in zoonotic digenetic trematodes may attribute to the environmental changes. The gaps in our knowledge of the epidemiology and control of zoonotic digenetic trematodes indicate the need for large cohort studies in most countries.

**Conclusions:**

This review provides important insights into the prevalence and global distribution of some zoonotic digenetic trematodes, firstly reveals spatio-temporal disparities in these digenetic trematodes. Countries with higher prevalence rate could be potential sources of transmitting diseases to other areas and are threat for possible outbreaks in the future. Therefore, continued global efforts to control and prevent human trematodiosis, and more international collaborations are necessary in the future.

**Graphical Abstract:**

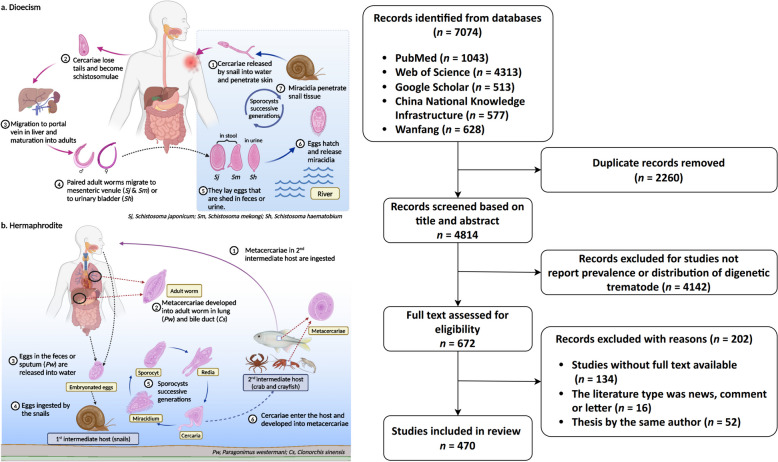

**Supplementary Information:**

The online version contains supplementary material available at 10.1186/s40249-024-01208-1.

## Background

Class Trematoda is consisted of subclasses Digenea, and Aspidogastrea [1], and flukes that parasitize in human are from subclass Digenea, which includes orders Strigeida (such as blood fluke), Echinostomida (such as intestinal fluke), and Plagiorchiida (such as liver fluke, lung fluke, and pancreatic fluke). Digenetic trematodes generally have similar physiological structures, characterized by dorsoventral compression, bilateral symmetry, and oral and ventral suckers [1]. All digenetic trematodes, except for the blood fluke, are hermaphrodites. Digenetic trematode is composed of integument and parenchyma, and possesses digestive, reproductive, excretory, and nervous systems [2].

**Fig. 1:**
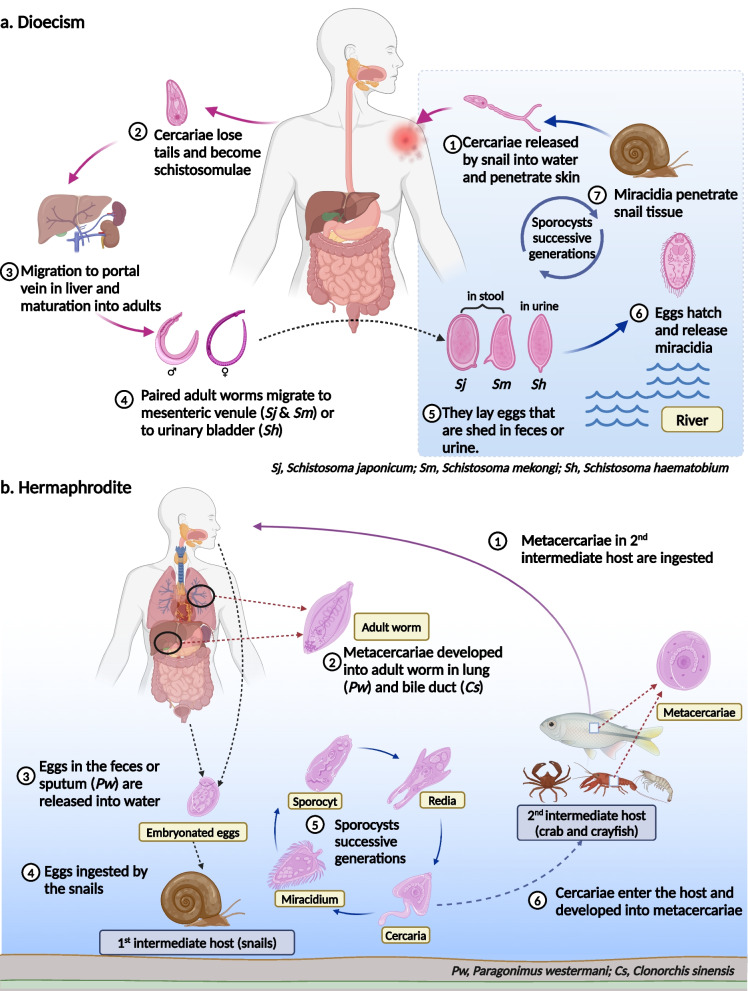
Life cycle of digenetic trematode. **a** Dioecism. The representative genus *Schistosoma* undergoes two stages in its life cycle, an asexual stage in snails and a sexual stage in mammals. Eggs are discharged into the water through feces (*Schistosoma japonicum* and *Schistosoma mansoni*) or urine (*Schistosoma haematobium*). Under appropriate conditions, the eggs hatch and release miracidia, which penetrate snail intermediate hosts. In snails, miracidia successfully complete sporocyst generations and produce the infective cercariae, which penetrate the skin of mammalian hosts (definitive hosts) and become schistosomulae. The schistosomulae migrate to lungs via venous circulation, then to the heart, and then develop in the liver, exiting the liver via the portal vein system when mature. Finally, adult worms copulate and reside in the mesenteric venules (*Schistosoma japonicum* and *Schistosoma mansoni*) or urinary bladder (*Schistosoma haematobium*). **b** Hermaphrodite. The representative genus *Clonorchis* and *Paragonimus* undergoes two stages in the life cycle, an asexual stage in snails and a sexual stage in mammals. Eggs in the feces (*Clonorchis* & *Paragonimus*) or sputum (*Paragonimus*) are discharged into water. Under appropriate conditions, the eggs hatch and release miracidia, which penetrate snail intermediate hosts. In snails, miracidia go through several developmental stages (sporocysts, rediae, and cercariae). The cercariae penetrate the flesh of raw fish or crabs, where they encyst as metacercariae. Mammalian hosts (definitive hosts) become infected by ingesting metacercariae on contaminated vegetables or in raw fish and crabs (foodborne trematodes). Finally, metacercariae developed into adult worm in lung (*Paragonimus*) and bile duct (*Clonorchis*). These figures are created with BioRender.com

The complex life cycle of digenetic trematodes involves asexual reproduction in the first intermediate hosts, and sexual reproduction in definitive hosts like humans and other vertebrates [3]. Commonly, the basic stages in the development process of digenetic trematodes include ovum, miracidium, sporocyst, redia, cercaria, encysted metacercaria, metacercaria, and adult (Fig. 1). Humans become infected by ingesting metacercariae in aquatic organisms including contaminated vegetables, raw fish and crabs (foodborne trematodes) or contacting cercariae in water (schistosomes). Since the hosts of digenetic trematodes are diverse, the transmission of trematodiosis in human is associated with the infection status in animal hosts [2]. Therefore, comprehensively grasp the species of hosts plays an important role in controlling this disease.

Because of the high diversities and wide distributions of digenetic trematodes, at least 200 million people affected by these flukes worldwide [4]. Therefore, it is crucial to have a better understanding of the epidemiology of trematodiosis. However, early reviews mainly focused on the distribution of one or several foodborne trematodes or schistosomes at that time [5–11], but ignored the spatio-temporal disparities in these digenetic trematodes. Therefore, in this scoping review, we conducted a comprehensive investigation on the spatio-temporal distribution of some important zoonotic digenetic trematodes, including species in *Schistosoma*, *Echinostoma*, *Isthmiophora*, *Echinochasmus*, *Paragonimus*, Opisthorchiidae, Fasciolidae, Heterophyidae, and *Eurytrema*, in order to provide useful insights for controlling and preventing human trematodiosis.

## Methods

### Search strategy, inclusion criteria and exclusion criteria

We searched the studies reporting digenetic trematodes using the PubMed (https://www.ncbi.nlm/nih.gov/pubmed/), Web of Science (https://www.webofscience.com/), Google Scholar (https://scholar.google.com), China National Knowledge Infrastructure (CNKI, https://www.cnki.net/), and Wanfang (https://www.wanfangdata.com.cn/) databases with no limits on the year of publication. Keywords in the search were the combinations of “trematode”, “fluke”, “digenea”, “epidemiology”, “distribution” and their expanded aspects, such as the generic name of digenetic trematode, continent name, country name and other identified search terms. The search terms used within five databases are listed in Additional file 1. The last retrieval time was December 2023.

Duplicated articles were initially removed by EndNote X9 (Clarivate, Philadelphia, USA), then, the titles and abstracts of the remaining articles were screened, those did not report prevalence or distribution of digenetic trematode were excluded. Full texts were evaluated carefully according to the following inclusion criteria: (1) the literature described the global distribution and epidemiology of one or more digenetic trematode aforementioned, (2) the literature type was article, review or case report. The exclusion criteria: (1) studies without full text available; (2) the literature type was news, comment or letter; (3) duplicates of graduate thesis by the same author. The search and selection processes of literatures were performed by two independent researchers, and any disagreement was mediated through consultation with a third researcher or team discussion until reaching a consensus.

### Quality assessment of included literature

The quality of the included articles was evaluated using the Joanna Briggs Institute Prevalence Critical Appraisal Tool [12]. The tool consists of ten quality control items, with each item assigned a score of either one or zero depending on its fulfillment. The scores were aggregated, where a total score of 0–3 indicates a low quality of the article, 4–6 a moderate quality, and 7–10 a high quality [13]. The quality assessment report of the included articles in this review is available in Additional file 2.

### Data extraction and analysis

All searched articles were processed by EndNote X9. The extracted data included: article title, author names, publication year, country of study, digenetic trematode species and its global distribution, prevalence, pathogenesis, and hosts. Subsequently, the extracted information was tabulated in Microsoft Excel 2016 (Microsoft Corp., Redmond, WA, USA) for descriptive analysis.

## Results

### Description of included studies

**Fig. 2:**
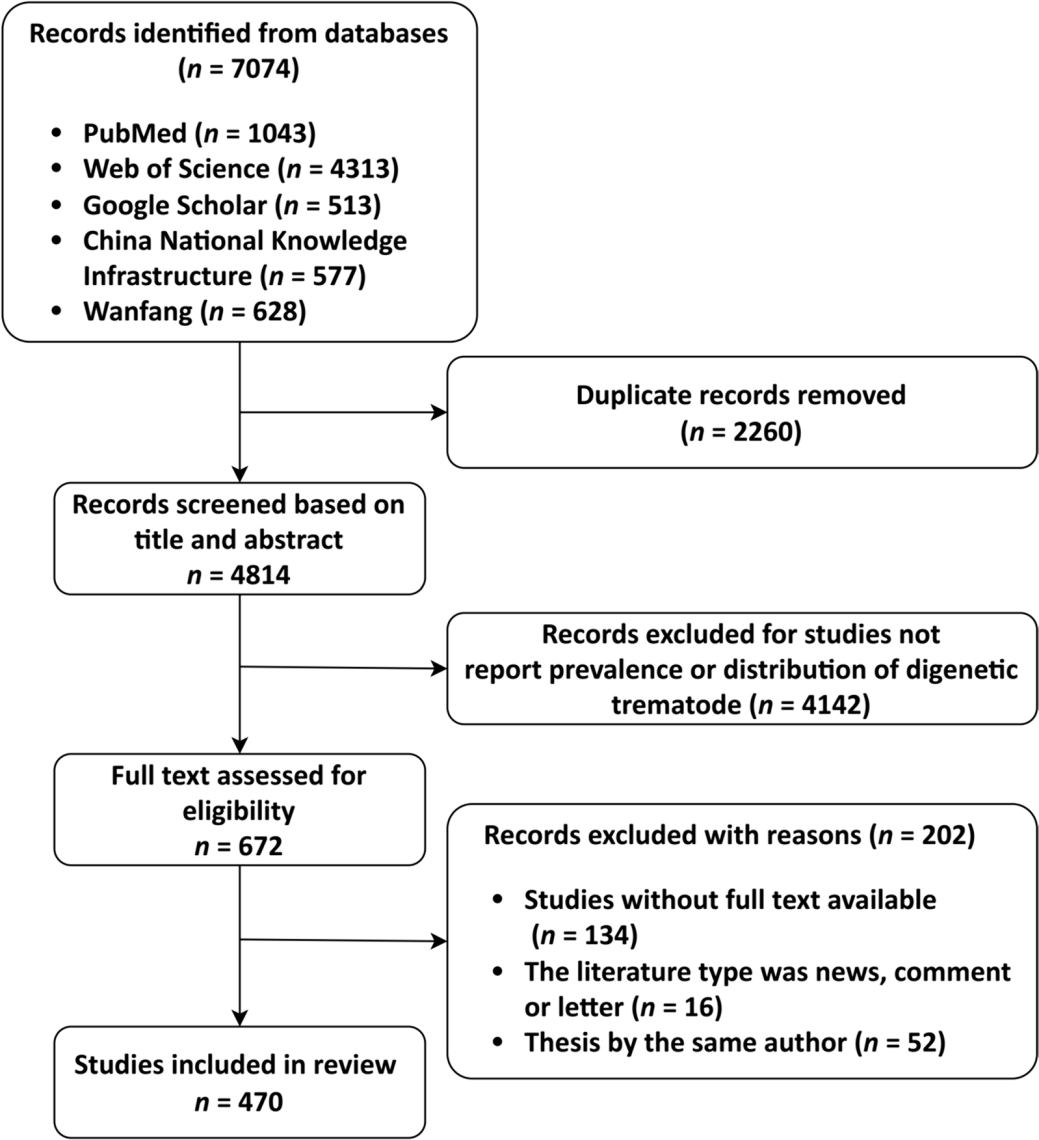
Flow diagram presenting the search process, including inclusion and exclusion criteria for articles screen

Based from the literature search, a total of 7074 articles were identified through database searching [PubMed (*n* = 1043), Web of Science (*n* = 4313), Google Scholar (*n* = 513), China National Knowledge Infrastructure (*n* = 577), and Wanfang (*n* = 628)]. After removal of duplicated records, 4814 articles were screened, 672 of which met the inclusion criteria. Following the full-text eligibility assessment, 470 articles were included in the review finally (Fig. 2). All literatures included were peer-reviewed articles. Moreover, Table 1 was created to exhibit the global distribution, temporal origins, and geographic origins of significant zoonotic digenetic trematodes in a more concise and organized manner.

**Table 1:** The global distribution, temporal and geographic origins of significant zoonotic digenetic trematodes

Genus	Species	Firstly discovered (year, country)	Epidemical range	1st intermediate host	2nd intermediate host	Definitive host	References
***Schistosoma***	*Schistosoma haematobium*	1851, Egypt	Africa, Middle East, Europe, Portugal, Mauritius, Mesopotamia, Madagascar, and India	*Bulinus* sp.		Various animals	[14–22]
	*Schistosoma mansoni*	1851, Egypt	Sub-Saharan Africa, Brazil, Venezuela, Suriname, Caribbean islands, Arabian, and Peninsula	*Biomphalaria pfeifferi* and* Australorbis glabratus*		Humans	[15, 17, 19–28]
	*Schistosoma japonicum*	1904, Japan	China, the Philippines, and Indonesia	*Oncomelania hupensis*		Various animals	[29–34]
	*Schistosoma mekongi*	1978, Cambodia	Several districts of Cambodia and Lao PDR	*Neotricula aperta*		Humans	[35–39]
***Echinostoma***	*Echinostoma revolutum*	1802, Germany	Asia (Bangladesh, China, India, Indonesia, Iran, Japan, Lao PDR, Malaysia, Republic of Korea, Thailand, and Vietnam), Oceania (New Zealand), Europe (Austria, Belarus, Bulgaria, Czech Republic, Finland, France, Germany, Greece, Hungary, Iceland, The Netherlands, Poland, Russia, Slovak Republic, UK, and Yugoslavia), and Americas (USA and Brazil)	Snails (families Planorbidae, Lymnaeidae, and Bulinidae)	Other snails, bivalves, fish, salamanders, and tadpoles	Birds, carnivores, rodents, and humans	[40–49]
***Isthmiophora***	*Isthmiophora hortensis*	1926, Japan	China, Republic of Korea, and Japan	Snails (families Planorbidae, Lymnaeidae, and Bulinidae)	Other snails, bivalves, fish, salamanders, and tadpoles	Birds, carnivores, rodents, and humans	[50, 51]
***Echinochasmus***	*Echinochasmus japonicus*	1926, Japan	Japan, China, Republic of Korea, Kuwait, Lao PDR, Russia, Thailand, and Vietnam	Snails (families Planorbidae, Lymnaeidae, and Bulinidae)	Other snails, bivalves, fish, salamanders, and tadpoles	Birds, carnivores, rodents, and humans	[5, 6, 52, 53]
	*Echinochasmus perfoliatus*	1902, Romania	China, Japan, Thailand, Republic of Korea, India, Vietnam, Denmark, England, Hungary, Russia, Poland, Ukraine, and Italy	Snails (families Planorbidae, Lymnaeidae, and Bulinidae)	Other snails, bivalves, fish, salamanders, and tadpoles	Birds, carnivores, rodents, and humans	[5, 54–57]
***Paragonimus***	*Paragonimus westermani*	1850, Brazil	China, the Philippines, Japan, Vietnam, Republic of Korea, Thailand, Malaysia, North Sumatra, and Indonesia	Freshwater snails	Crustaceans	Mammals	[58–66]
	*Paragonimus skrjabini*	1959, China	China, India, Indonesia, Iran, Japan, Lao PDR, Malaysia, Republic of Korea, Vietnam, Austria, Russia, United Kingdom, and Americas	Freshwater snails (mollusk)	Crustaceans	Dogs, cats, or humans	[67–71]
**Opisthorchiidae**	*Clonorchis sinensis*	1874, India and Mauritius	China, Republic of Korea, Vietnam, and Russia	Freshwater snails (families Bithyniidae, Pleuroseridae, Assimineidae, and Thiaridae)	Freshwater fishes	Humans, domestic canids and felids, swine, mustelids, and other piscivorous mammals	[6, 72, 73]
	*Opisthorchis felineus*	1892, Russia	Germany, Greece, Poland, Romania, Italy, Spain, Belarus, Ukraine, Kazakhstan, and Russia	Snails (*Bithynia inflata*, *Bithynia troschelii*, *Bithynia leachii*, and *Bithynia tentaculata*)	Freshwater fishes (*Leuciscus idus*, *Tinca tinca*, and *Abramis brama*)	Cats, dogs, and various fish-eating mammals including humans	[1, 6, 73–75]
	*Opisthorchis viverrini*	1886, France	Thailand, Lao PDR, Vietnam, Cambodia, Malaysia, and Myanmar	*Bithynia goniomphalus, B. funiculate*, and *B. siamensis*	Cyprinoid fish	Cats, dogs, and various fish-eating mammals including humans	[6, 73, 74, 76]
	*Metorchis orientalis*	1920, Japan	East Asia (China, Japan, and Republic of Korea)	*Parafossarulus* sp.	Cyprinoid fish	Dogs, cats, ducks, chickens, and geese	[77–81]
**Fasciolidae**	*Fasciola hepatica*	1379, France	Ecuador, Bolivia, Chile, Peru, Cuba, Egypt, Portugal, France, Spain, Iran, Turkey, Republic of Korea, Japan, China, Thailand, Vietnam, Switzerland, Sweden, Portugal, Norway, England, Wales, Scotland, Denmark, Germany, Brazil, Pakistan, Colombia, Costa Rica, Poland, Mexico, Ghana, Australia, Ethiopia, Estonia, Tanzania, the United States, Argentina, Haiti, and Kyrgyzstan	Lymnaeidae		Ruminants and non-ruminant herbivores	[7, 73, 82–89]
	*Fasciola gigantica*	1855, Pakistan	Indonesia, Cambodia, Thailand, the Philippines, Vietnam, China, Burma, Pakistan, India, Nepal, lran, Egypt, Sudan, Tanganyika, Malawi, Chad, Mali, Kenya, Tanzania, Nigeria, Cameroons, West Africa, Zambia, Zimbabwe, Uganda, and Ethiopia	*Galba truncatula*, *Radix natalensis*, and *Pseudosuccinea columella*		Humans and livestock	[83, 90–94]
	*Fasciolopsis buski*	1843, India	China, Thailand, Vietnam, Malaysia, Myanmar, Indonesia, India, and Lao PDR	Snails (*Segmentina* sp. and *Hippeutis*)		Humans and pigs	[95–103]
**Heterophyidae**	*Heterophyes heterophyes*	1851, Egypt	Egypt, Tunisia, Iran, Middle East, Republic of Korea, Egypt, Sudan, and Japan	Snails (*Cerithidia* sp. and *Pironella* sp.)	Fresh/brackish water fishes	Humans, various fish-eating mammals (e.g., cats and dogs), and birds	[6, 104–108]
	*Heterophyes nocens*	1916, Japan	Japan, Republic of Korea, China, and Thailand	Brackish water snail (*Cerithidea* sp.)	Brackish water fishes (mullet and goby)	Humans, cats, and dogs	[109–112]
	*Haplorchis pumilio*	1896, Egypt	Asia, Oceania, America, Vietnam, Thailand, Egypt, and China	*Thiara tuberculata*	Freshwater fishes	Dogs, cats, birds, and humans	[6, 113, 114]
	*Haplorchis taichui*	1924, China	The Philippines, Bangladesh, India, Sri Lanka, Palestine, Iraq, Egypt, Malaysia, Thailand, Lao PDR, Vietnam, and South China	Snails (*Melania obliquegranosa*, *Stenomelania juncea*, and *Melanoides tuberculate*)	Freshwater fishes	Dogs, cats, birds, and humans	[115–118]
	*Metagonimus yokogawai*	1912, Japan	Far Eastern Russia, Republic of Korea, Japan, and China	Semisulcospiridae snails	Fresh/brackish water fishes	Humans, fish-eating mammals (e.g., cats and dogs), and birds	[77, 119–122]
	*Stellantchasmus falcatus*	1916, Japan	Hawaii, Republic of Korea, and Vietnam	Brackish water snails	Mullet	Humans, rats, cats, dogs, and chickens	[50, 123–125]
	*Centrocestus formosanus*	1985, China	China, Japan, Thailand, USA, Brazil, Lao PDR, Vietnam, Thailand, and Mexico	*Melanoides tuberculatus*	Freshwater fishes, frogs, and toads	Local piscivorous birds	[7, 126–133]
***Eurytrema***	*Eurytrema cladorchis*	1965, China	China, Nepal, Bangladesh, Indonesia and Vietnam	*Bradybaena similaris*	*Nemobius* sp.	Domestic and wild ruminants and humans	[134–137]

The included studies were carried out in Asia (39.4%; *n* = 185), Africa (20.0%; *n* = 94), North America (9.8%;* n* = 46), South America (7.0%; *n* = 33), Europe (20.9%; *n* = 98), Oceania (3.0%; *n* = 14), and Antarctica (0.0%; *n* = 0).

**Fig. 3:**
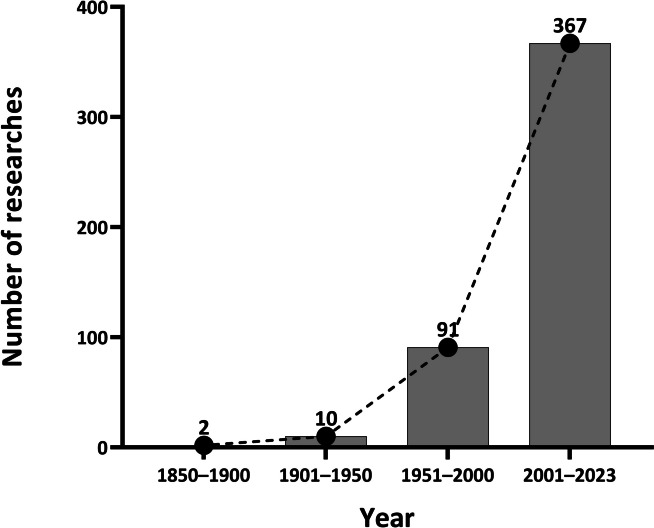
Total number of the research articles searching through PubMed, Web of Science, Google Scholar, CNKI and Wanfang databases

Moreover, a comprehensive historical analysis of the published research has been conducted across distinct time periods: 1850–1900, 1901–1950, 1951–2000, and 2001–2023, encompassing 2, 10, 91, and 367 studies conducted in each respective era (Fig. 3).

### Global distribution and epidemiology

#### Schistosoma

##### Global distribution

*Schistosoma haematobium* was first discovered in 1851 in Egypt by Bilharz during a necropsy [14, 15]. Subsequently, *S. haematobium* was found in Egypt and Iraq between 1901 and 1950 [16] (Additional file 3: Fig. S1a). Whereafter, *S. haematobium* was reported in Africa, the Middle East, and India from 1951 to 2000 [17] (Additional file 3: Fig. S1b). At present, *S. haematobium* is prevalent in Africa, the Middle East, and Europe [18]. Although Europe is not an endemic area, *Schistosoma* has been introduced through migration and travel [19]. In sub-Saharan Africa, *S. haematobium* is mostly distributed in Nigeria [20], Volta basin, Ghana, southwest Cameroun [21] and Mozambique [22]. Furthermore, it occurs in Portugal, Mauritius, Mesopotamia and Madagascar. A few cases have been reported in Mumbai and India (Additional file 3: Fig. S1c).

In 1902, Manson found eggs with lateral spine in the feces of an English patient [15]. Sambon proposed this new species named after *Schistosoma mansoni* in 1907 [15] (Additional file 3: Fig. S1a). From 1951 to 2000, *S. mansoni* was reported in the Middle East [23], Africa, Brazil, Venezuela, and the Caribbean [17] (Additional file 3: Fig. S1b). *S. mansoni* is primarily distributed in sub-Saharan Africa, the Middle East, some South American countries (Brazil, Venezuela, and Suriname), and the Caribbean islands [24], with sporadic reports in the Arabian Peninsula [23] and a few European countries [19]. In sub-Saharan African region, *S. mansoni* is mostly distributed in Ethiopia [25], Nigeria [20], Tanzania (Sengerema District, Nyamatongo Ward), north Ghana [21], Mozambique [22], Rwanda [26], and Democratic Republic of the Congo [27] (Additional file 3: Fig. S1c). Recently, a systematic review and meta-analysis in Ethiopia showed that the distribution area of *S. mansoni* exhibit environmental and ecological heterogeneities, where the soil’s silt and clay contents are higher than 22.0% [28].

In 1903, Kawanishi first discovered trematode eggs in stool examination of a patient. On May 30, 1904, Fujinami discovered a female parasite in the portal vein when performing an autopsy (Additional file 3: Fig. S1a). He designated it as a new species, *Schistosoma japonicum* [29]*.* From 1951 to 2000, cases of *S. japonicum* infection were reported in China, Japan, and the Philippines [30] (Additional file 3: Fig. S1b). *S. japonicum* used to be endemic in Japan, but was eliminated in 1996 [31]. Currently, it is mainly distributed in East Asia and Southeast Asia, including China, the Philippines [32], and a few regions of Indonesia such as Sulawesi [33, 34] (Additional file 3: Fig. S1c).

Schistosomiasis was initially reported in the Mekong River's Lower Basin region in 1957, specifically from the Laotian island of Khong to the Cambodian province of Kratie. At that time, *S. japonicum* was believed to be the cause, until a major revelation in 1978. This was when *Neotricula aperta* was discovered and *Schistosoma mekongi*, a unique species, was identified for the first time as the true cause of these cases [35–37] (Additional file 3: Fig. S1b). To date, *S. mekongi* is a restricted *Schistosoma* species found near the Mekong River, mainly in southern Lao PDR and northern Cambodia [38, 39] (Additional file 3: Fig. S1c).

##### Epidemiology

According to World Health Organization (WHO), an estimated 240 million people worldwide are infected with *S. haematobium*, while 90 million people are infected with *S. mansoni*, and 25 million people are infected with *S. japonicum* [40]. Approximately, 140,000 people are subject to the risk of infection by *S. mekongi*, with 80,000 found in Cambodia and a further 60,000 in Lao PDR [41]. Despite the number appearing small, continuous infection and re-infection contribute to the persistence of the disease within these susceptible populations. Notably, children are the most impacted due to their greater engagement with water [42]. Preventive measures for schistosomiasis include health education, improved water supply and sanitation, and mass drug administration of praziquantel, which is the recommended treatment for *Schistosoma* infection. Control and elimination programs for schistosomiasis are ongoing in many endemic countries.

#### *Echinostoma* and *Isthmiophora*

##### Global distribution

*Echinostoma revolutum*, the type species of the genus *Echinostoma*, was first reported by Froelich in Germany in 1802. However, the first reported human infection occurred in Taiwan province, China in 1929 [43] (Additional file 4: Fig. S2a). Between 1951 and 2000, *E. revolutum* was reported in China [44], Australia, New Zealand [45], USA [46], and Thailand [47] (Additional file 4: Fig. S2b). Currently, *E. revolutum* is the most widely distributed species among the known *Echinostoma* species and can be found in Asia (Bangladesh, China, India, Indonesia, Iran, Japan, Lao PDR, Malaysia, Republic of Korea, Thailand, and Vietnam), Oceania (New Zealand), Europe (Austria, Belarus, Bulgaria, Czech Republic, Finland, France, Germany, Greece, Hungary, Iceland, The Netherlands, Poland, Russia, Slovak Republic, UK, and Yugoslavia), and Americas (USA and Brazil), but rarely found in Africa [45, 46, 48–51]. However, reports in recent years show that outbreaks have been reported in North America after travellers returned from Eastern Africa including Kenya and Tanzania [52] (Additional file 4: Fig. S2c).

Another important species, *Isthmiophora hortensis*, was first reported by Asada in Japan in 1926 (Additional file 4: Fig. S2a). In 1964, *I. hortensis* was reported in Republic of Korea [53] (Additional file 4: Fig. S2b). The current global distribution of *I. hortensis* is primarily localized to East Asia, especially in China, Republic of Korea, and Japan [54] (Additional file 4: Fig. S2c).

##### Epidemiology

In 2004, the WHO estimated the number of human infection for *I. hortensis* was approximately 50,000 [40]. The prevalence of *E. revolutum* among people in Taiwan province, China was estimated to be 2.8–6.5% [6]. Infection rates of *E. revolutum* were found to range from 7.5% to 22.4% among schoolchildren in Pursat Province, Cambodia [6]. However, the number of individuals currently infected or at risk of echinostomiasis remains unclear [55], highlighting the need for further research in this area.

*Echinostoma* are commonly found among birds and mammals in fresh water habitats, contributing to their ubiquitous presence. Echinostomiasis tend to be foci of infection in places where raw or undercooked intermediate hosts are eaten, so the most effective measure to prevent human infection is to eliminate the consumption of raw or undercooked freshwater snails, clams, fishes, or amphibians [5].

#### Echinochasmus

##### Global distribution

*Echinochasmus japonicus* was first reported in the experimental infection of dogs, cats, rats, mice and birds in Japan in 1926 [5] (Additional file 5: Fig. S3a). Natural infections of *E. japonicus* were reported in China [56] and Republic of Korea [57] from 1951 to 2000 (Additional file 5: Fig. S3b). Human infection of *E. japonicus* is mostly restricted in Asia. It has been reported in Japan, China, Republic of Korea, Kuwait, Lao PDR, Russia, Thailand, and Vietnam [6] (Additional file 5: Fig. S3c).

*Echinochasmus perfoliatus* was first identified in dogs in Romania by Motas and Straulescus in 1902 and was found in cats and dogs in Hungary by Ratz in 1908 (Additional file 5: Fig. S3a). In 1998, it was discovered in the Far East of Russia [58] (Additional file 5: Fig. S3b). Moreover, the natural and experimental infection in human was first found in Japan [59]. The epidemiology of *E. perfoliatus* infection is not only restricted in Asian area including China, Japan, Thailand, Republic of Korea, India, Vietnam, and the Philippines [5]. Cases or studies concerning the prevalence in the intermediate or definitive hosts were also reported from European countries like Denmark, England, Hungary, Russia, Poland, Ukraine, and Italy [5]. An infection of *E. perfoliatus* in gastrointestinal tract has been reported in Egypt on white ibis recently [60]. And similar finding was shown in Denmark on red foxes, another common reservoir host of *E. perfoliatus* [61] (Additional file 5: Fig. S3c).

##### Epidemiology

As for *E. japonicus*, a larger-scale remote study conducted in Fujian and Guangdong province, China reported the prevalence rates in people, dogs and cats as 4.9%, 39.7% and 9.5%, respectively [59]. Furthermore, a study conducted in Lao PDR reported an overall infection rate in human was 3.1% [62]. Additionally, research has been conducted on the infection of *E. japonicus* in stray cats in various countries including Kuwait (1.6%) [63], and Republic of Korea (2.6% along the Geumgang river) [64]. Furthermore, a prevalence rate of 1.8% was reported for *E. perfoliatus* among individuals residing in the Guangdong, Fujian, Anhui, and Hubei provinces of China [6].

Like other reservoir hosts, including dogs, cats, ducks, and birds, humans are infected by eating the insufficiently cooked aquatic animals with encysted metacercariae. Hence, proper cooking of fish and improved sanitation and hygiene practices are essential to avoid the infection. Praziquantel is recognized as the first-line drug while albendazole as an alternative treatment option.

#### Paragonimus

##### Global distribution

*Paragonimus westermani* is the most prominent species among the genus *Paragonimus* [65]. The first report on *P. westermani* could date back to 1877, when Kerbert detected it in the lungs of a Bengal tiger [66]. Between 1951 and 2000, *P. westermani* was reported in Japan, China, Republic of Korea, the Philippines, peninsular Malaysia, and Thailand [67] (Additional file 6: Fig. S4a). Currently, paragonimiasis (lung fluke disease), which is caused by the parasitic flatworm, is endemic to several parts of Asia, Africa, South America, and North America, but occurs primarily in China, the Philippines, Japan, Vietnam, Republic of Korea, Thailand, Malaysia, North Sumatra, and Indonesia [65, 67–73] (Additional file 6: Fig. S4b).

The first identified specimens of *Paragonimus skrjabini* were obtained from the lungs of a viverrid, *Paguma larvata*, located in Guangzhou city, Guangdong Province, China in 1959 [74] (Additional file 6: Fig. S4a). Initially, the species was classified as *Paragonimus szechuanensis* when the first report of infection occurred in Sichuan Province, China. Subsequently, further research revealed that *P. szechuanensis* was synonymous with *P. skrjabini* [75]. It is commonly found in Asia (China, India, Indonesia, Iran, Japan, Lao PDR, Malaysia, Republic of Korea, and Vietnam), Europe (Austria, Russia, and UK) and Americas [76–78] (Additional file 6: Fig. S4b).

##### Epidemiology

An estimated 293.8 million individuals worldwide are at risk of *Paragonimus* infection, with China being the most heavily affected country, accounting for 195 million cases. Notably, *P. westermani* has been identified in multiple provincial-level administrative divisions across China, including Guangdong, Fujian, Yunnan, Guangxi, Guizhou, Hubei, Jiangxi, Hunan, Henan, Shaanxi, Gansu, Zhejiang, Sichuan, Hunan, Hainan, and Shanxi [78].

In certain endemic areas, the prevalence of paragonimiasis can be as high as 10% or more. To prevent the disease, it is recommended to cook crabs thoroughly and improve sanitation and hygiene practices. Praziquantel or triclabendazole are typically used as treatment for paragonimiasis.

#### Opisthorchiidae

##### Global distribution

*Clonorchis sinensis* is the third most prevalent human fluke globally. The first reports of *C. sinensis* were made in 1874, nearly at the same time by MacConnell in India and MacGregor in Mauritius [79]. Between 1901 and 1950, reports of *C. sinensis* were documented in China and Japan [79] (Additional file 7: Fig. S5a). Furthermore, *C. sinensis* was reported in Republic of Korea, Vietnam, and the Far East of Russia between 1951 and 2000 [80] (Additional file 7: Fig. S5b). Nowadays, the endemic areas are mainly located in the Far East and East Asia, such as China, Republic of Korea, Vietnam, Russia, excluding Japan [6] (Additional file 7: Fig. S5c).

*Opisthorchis felineus* was first reported in the liver of cats, and human infection was first reported by Winogradoff in Tomsk, Siberia in 1892 [1, 81]. Between 1951 and 2000, it was reported in Kazakhstan, Italy, Albania, Greece, Switzerland, Holland, Germany, Poland, Ukraine, Bielorussia, Turkey, and Siberia [80] (Additional file 7: Fig. S5b). Eggs of *O. felineus* were found in fecal fossils of human and dog in Russia [82]. Nowadays, it’s worldwide prevalent in Germany, Greece, Poland, Romania, Italy, Spain, Belarus, Ukraine, Kazakhstan, and Russia [6] (Additional file 7: Fig. S5c).

*Opisthorchis viverrini* was first detected in the liver of *Felis viverrini*, a civet cat brought from India to France in 1886 and Leiper reported Jiang's first human infection through autopsy of two prisoners in 1991 [81, 83]. Between 1951 and 2000, *O. viverrini* was reported in Thailand, Lao PDR, Cambodia, and Malaysia [80] (Additional file 7: Fig. S5b). Currently, it’s also mainly prevalent in the southeast Asian countries such as Thailand, Lao PDR, Vietnam, Cambodia, Malaysia, and Myanmar [6] (Additional file 7: Fig. S5c).

*Metorchis orientalis* was first detected in *Anas platyrhynchos domestica* (ahiru) by Tanabe in Fukuyama city in Japan in 1920 [84] (Additional file 7: Fig. S5a). *M. orientalis* is a liver fluke species that infects piscivorous birds and mammals, including humans in East Asia [85–87]. The first documentation of human infections dates back to 2001, when 4 (4.21%) out of 95 residents examined in Ping Yuan County of Guangdong Province, China, were found to be infected. Furthermore, 12 adult flukes were retrieved from two purged patients [85]. The geographical range of *M. orientalis* appears to overlap with that of *C. sinensis*, mainly distributed in East Asia including China, Japan, and Republic of Korea until now [7, 88] (Additional file 7: Fig. S5c).

##### Epidemiology

Previous reports have revealed that the prevalence of opisthorchiasis, the disease caused by *Opisthorchis*, can reach up to 70% or more in some endemic areas [89]. In Asia, *C. sinensis* is currently the most prevalent parasite, infecting approximately 15 million people, with an estimated 200 million at risk of persistent infection. Moreover, at least 1.6 million people are infected with *O. felineus* of the total of 17 million infested with the *Opisthorchis* flukes [90], while the estimated number of people infected with *O. viverrini* is 9–10 million [7].

Appropriate cooking of freshwater fish, as well as improved sanitation and hygiene practices, are effective preventive measures for opisthorchiasis. Praziquantel or albendazole are generally applied to the treatment for the disease.

#### Fasciolidae

##### Global distribution

Currently, *Fasciola hepatica* has a widely distributed geographical range among parasitic and vector-borne diseases. Reports of liver fluke could date back to 1379 in France [91], but it was not until 1523 that Fitzherbert published the first detailed description of it. *F. hepatica* is widely believed to have originated in Eurasia [80], specifically in the Near East of Asia [91, 92]. Later, *F. hepatica* has spread west into Europe [93], east into Asia [94], south into Africa [95], and transocetically into Oceania and Americas [96] (Additional file 8: Fig. S6b). Numbers of clinical cases of *F. hepatica* reported have been increasing since 1970 [80]. Nowadays, *F. hepatica* is found on all inhabited continents, in more than 70 countries. Human infections have been reported in Ecuador, Bolivia, Chile, Peru, Cuba, Egypt, Portugal, France, Spain, Iran, Turkey, Republic of Korea, Japan, China, Thailand, and Vietnam [7, 97, 98] (Additional file 8: Fig. S6c).

Another species responsible for fascioliasis is *Fasciola gigantica*, which was described by Cobbold from the liver of the giraffe in Pakistan in 1855 [99]. *F. gigantica* is limited to the tropical and subtropical regions of Africa, Asia and the Far East where *Radix* vectors allow for their transmission [92]. In the twentieth century, *F. gigantica* was reported in Indonesia, Cambodia, Thailand, the Philippines, Vietnam, China, Burma, Pakistan, India, Nepal, Iran, Egypt, Sudan, Tanganyika, Malawi, Chad, Mali, Kenya, Tanzania, Nigeria, Cameroons, West Africa, Zambia, Zimbabwe, Uganda, Ethiopia [100] (Additional file 8: Fig. S6a and S6b). At present, *F. gigantica* is the main species responsible for fasciolosis in Cambodia [101], Lao PDR [102], China [103] and Thailand [103] (Additional file 8: Fig. S6c).

*Fasciolopsis buski* is the largest trematode in the world, which was first discovered in the duodenum of an Indian sailor in 1843 [104, 105]. Lankester named it *Distoma buski* in 1857. Later, the species was transferred to the genus *Fasciolopsis* by Odhner. In the last century, fasciolosis was prevalent in China [106] (Additional file 8: Fig. S6a). Its presence in India was reported in 1972 [107] (Additional file 8: Fig. S6b). *F. buski* is of Asian origin and mainly distributed in China [106], Southeast Asia (Thailand, Vietnam, Malaysia, Myanmar, and Indonesia) [108–110], and India [111, 112] (Additional file 8: Fig. S6c).

##### Epidemiology

The prevalence of fascioliasis in certain endemic areas can be as high as 90% or more [97]. Approximately 2.6 million individuals are estimated to be infected with *Fasciola* spp., based on limited country prevalence data and expert opinion [113]. *F. gigantica* is of greatest importance as a parasite of cattle and buffalo although, there are occasional reports of human infection with *F. gigantica*, mainly case studies [100]. An estimated minimum of 10 million people in Asia are infected with *F. buski* [114]*.* The prevalence of *F. buski* among human populations varies, ranging from 0.04% in Cambodia to 8.6% to 50% in Bangladesh, up to 85% in certain regions of China [6].

Therefore, cooking aquatic plants properly as well as improving sanitation and hygiene practices are the key measures to avoid being infected. Treatment for the disease typically involves the use of praziquantel or bithionol.

#### Heterophyidae

##### Global distribution

*Heterophyes heterophyes* was initially discovered in the human intestine by Bilharz in Egypt in 1851 [115]. From 1951 to 2000, it was reported in Egypt, the Middle East [116], Republic of Korea [117], and Japan [118] (Additional file 9: Fig. S7b). Currently, it is widely distributed from Europe to the Middle East and North Africa, particularly in Egypt, Tunisia, and Iran [6]. Cases of human infection have been reported in various countries, including Republic of Korea [116], Egypt [119], Sudan [117], Japan [118], and so on (Additional file 9: Fig. S7c).

Another species of *Heterophyes*, *Heterophyes nocens*, was first discovered by Onji and Nishio in Japan in 1916 [120] (Additional file 9: Fig. S7a). It was reported in Republic of Korea in 1981 [121], in China in 1994 [122] (Additional file 9: Fig. S7b). Nowadays, there were also some cases in Thailand since 2015 [123] (Additional file 9: Fig. S7c).

*Haplorchis pumilio* was first discovered as a natural infection in birds in Egypt in 1899 [124]. However, 12 cases of human infection have been reported in Thailand in 1983 [125] (Additional file 10: Fig. S8b). Nowadays, *H. pumilio* has been identified in various regions spanning Africa, Asia, Oceania, and America, with higher prevalence rates observed in Vietnam and Thailand [6] (Additional file 10: Fig. S8c).

*Haplorchis taichui* was first identified in Taiwan Province, China in 1924 from birds and mammals [126] (Additional file 10: Fig. S8a). Natural human infections were first reported in the Philippines [127]. Currently, it is mainly distributed in Asia (the Philippines, Malaysia, Thailand, Lao PDR, Vietnam, China, Bangladesh, India, and Sri Lanka) and the Middle East (Palestine, Iraq, and Egypt) [128, 129] (Additional file 10: Fig. S8c).

*Metagonimus yokagawai*, which was found in Japan in 1912 for the first time [84] (Additional file 11: Fig. S9a). From 1951 to 2000, it was reported in Republic of Korea [130] and Japan (Additional file 11: Fig. S9b). Currently, it has been found parasitizing mammals and birds in Republic of Korea, Japan, and China [131] (Additional file 11: Fig. S9c). Although no human infections have been confirmed, numerous epidemiological investigations have identified that high-risk endemic areas are primarily located along rivers [130, 132, 133].

*Stellantchasmus falcatus* was first observed in experimentally feeding cats in Japan in 1916 [134]. Subsequently, there were reports of human infections in Hawaii in 1938 [135] (Additional file 11: Fig. S9a), followed by cases reported in Republic of Korea in 1990 [53] (Additional file 11: Fig. S9b), as well as in Vietnam [136], and several other countries, particularly in Asia (Additional file 11: Fig. S9c).

*Centrocestus formosanus* was first identified in Taiwan Province, China in 1924 [137] (Additional file 11: Fig. S9a). Between 1951 and 2000, it was reported in China [138], Thailand [139], Malaysia [140], and Mexico [141] (Additional file 11: Fig. S9b). At present, *C. formosanus* distribute widely in Asia and America*,* covering China, Japan, Lao PDR [142], Vietnam [143], Thailand [144], USA, Brazil, Mexico and so on [7, 141] (Additional file 11: Fig. S9c).

##### Epidemiology

A cross-sectional study conducted on 996 randomly selected preschool and school-aged children in Gharbia governorate during January to April 2018 showed that the prevalence of *H. heterophyes* is 1.4% [119]. In Republic of Korea, residents of southwestern coastal areas and islands showed a 10–70% positive rate for *H. nocens* [145]. In Republic of Korea, the national average prevalence of heterophyid eggs, mainly *M. yokogawai*, was 0.5% in 2004 and the estimated number of infected individuals in Republic of Korea is approximately 260,000 [146]. In Japan, the reported prevalence of *M. yokogawai* in humans had been 0.5–35.1% until the 1960s depending on the locality [6]. In Vietnam, between January 2009 and December 2010, the stool of a total of 405 people with the habit of eating raw fish were collected for examination of the presence of fish-borne trematodes, revealing a 52.08% prevalence for *H. pumilio* and 1.04% prevalence for *C. formosanus* [139]. In Republic of Korea, although only 4 cases of *S. falcatus* infection in humans have been confirmed, the estimated number of human cases is 5000 [57]. An epidemiological survey indicates that the raw estuarine fish consumption is linked to the prevalence of *S. falcatus* infections among residents in endemic areas [147], therefore, improving the dietary habits of these populations is critical. The prevalence of *H. taichui* infection is high in certain regions of Southeast Asia, with reported cases reaching 4,138,169; in these areas, the eggs of this parasite are frequently mistaken for those of *Opisthorchis viverrine* [148].

Humans get infected by consuming raw or undercooked freshwater fish or crustaceans that contain metacercariae. Therefore, improving the dietary habits of residents in endemic areas is critical. Treatment for heterophyidiasis typically involves the use of praziquantel or albendazole.

#### Eurytrema

##### Global distribution

*Eurytrema cladorchis* was first described from pancreatic duct of wild deer in the mountain area of Guizhou Province in China in 1965 [149] (Additional file 12: Fig. S10a). Existence of the flukes was first reported from domestic ruminants in Nepal in 1985 [150] (Additional file 12: Fig. S10a). At present, *E. cladorchis* was found in cattle in Bangladesh [151], Indonesia [152] and Vietnam [150] (Additional file 12: Fig. S10b).

##### Epidemiology

*E. cladorchis* infection is endemic in livestock in the mountain villages in China bordering Fujian, Zhejiang and Jiangxi provinces. From 83 to 100% of cattle were found to be infected with as many as 542 to 1840 flukes/animal [153].

## Discussion

Our analysis revealed that the distribution of zoonotic digenetic trematodes is geographically widespread, with certain species being more prevalent in specific regions. For instance, species within *Schistosoma*, including *S. haematobium*, *S. mansoni*, and *S. japonicum*, are primarily found in Africa, the Middle East, and Asia [16, 22–24]. The prevalence of *Schistosoma* infections is substantial, with millions of people affected worldwide, underscoring the significant burden of schistosomiasis on public health [154]. Similarly, other digenetic trematodes such as *Echinostoma*, *Isthmiophora*, and *Echinochasmus* exhibit specific geographic distributions, with varying prevalence rates in different regions [54, 155]. Transmission typically occurs through consumption of undercooked aquatic animals, pointing to the critical role of safe food handling practices in infection prevention. The prevalence of these trematodes varies across different endemic areas, emphasizing the need for region-specific control strategies and surveillance efforts. Furthermore, this review confirms that digenetic trematodes are widespread globally and are a significant problem in many regions, particularly in developing countries in Asia and Africa. However, even countries without endemic digenetic trematodes are at risk due to increased global travel and migration [1–4]. This underlines the need for a global approach to control and prevention of these infections. The research further underscores the imperative for expanded studies on infection control and treatment. Despite praziquantel's efficacy in treating numerous trematodiasis cases, there are instances of drug-resistant flukes [156–158]. Thus, comprehensive infection control strategies must go beyond medical treatment, emphasizing hygiene, sanitation improvement, and possibly biological snail control.

Although our study has updated current knowledge on the spatio-temporal distribution of several zoonotic digenetic trematodes, there are still considerable gaps in our understanding. First, many studies identified were case reports or small-scale studies, possibly underrepresenting the actual infection burden. Additionally, research concentration in Asia and Africa might denote higher prevalence or reflect underreporting and surveillance deficits in less scrutinized locales. Moreover, the complex life cycles of these trematodes, involving multiple hosts, complicate the comprehension of their transmission dynamics, necessitating broad-based global collaboration for effective control and prevention strategies. Hence, there is an urgent call for increased researches and intervention efforts, particularly in developing countries disproportionately affected by these digenetic trematodes. Continued implementation of effective control tactics and improvements in health education are critical moving forward. Moreover, fostering cooperation among various stakeholders, including parasitologists, veterinarians, medical professionals, public health officials, and policymakers, is essential to ensure comprehensive and effective disease management strategies.

## Conclusions

Trematodiosis, which is caused by digenetic trematodes, constitutes a severe public health and economic concern worldwide. In this review, we provided important insights into some zoonotic digenetic trematodes with respect to the prevalence and global distribution, firstly revealed spatio-temporal disparities in these digenetic trematodes. Through the distribution of zoonotic digenetic trematodes from the same genus, we found some overlaps between them, which indicated the risk of co-infections that could increase transmission. Moreover, the prevalence and global distribution of zoonotic digenetic trematodes did not equate, the former relies on various factors, including the detection methods and test population. Obviously, the source of infection, route of transmission, and susceptible population are key factors of trematodiosis prevalence, countries with higher prevalence rate could be potential sources of transmitting diseases to other areas and are threat for possible outbreaks in the future. Recent global changes such as climate warming, environmental alterations, changes in dietary patterns, and increased international travel and cooperation have contributed to the spread of trematodiosis. The temporal disparities in zoonotic digenetic trematodes may attribute to environmental temperature, precipitation and the population dynamics of hosts. Therefore, improved diagnostic methods, effective treatment of patients, host control, and raising public health awareness, even in developed areas, are key factors in safeguarding public health from trematodiosis. Furthermore, this review calls for continued global efforts to control and prevent human trematodiosis, and more international collaborations are necessary in the future.

However, despite the unquestionable progress achieved in the prevalence of trematodiosis, epidemiological data are lacking in most countries, emphasizing the need for large cohort studies. Also, there is an urgent need to improve the pathogenesis, applying omics technologies in research would enable a more comprehensive understanding of fluke biology, physiology, and genetics as well as mechanisms during trematodiosis development. This could potentially reveal new targets for early diagnosis, treatment, and prognosis to support the prompt elimination of trematodiosis.

### Supplementary Information

Additional file 1: Search strategies in each database.

Additional file 2: Quality assessment report of included articles in this review.

Additional file 3: Fig. S1. Global distribution of* Schistosoma haematobium*, *Schistosoma mansoni*, *Schistosoma japonicum*, and *Schistosoma mekongi*. (a) 1901–1950. (b) 1951–2000. (c) 2001–2023.

Additional file 4: Fig. S2. Global distribution of *Echinostoma revolutum* and *Isthmiophora hortensis*. (a) 1901–1950. (b) 1951–2000. (c) 2001–2023.

Additional file 5: Fig. S3. Global distribution of *Echinochasmus japonicus *and* Echinochasmus perfoliatus*. (a) 1901–1950. (b) 1951–2000. (c) 2001–2023.

Additional file 6: Fig. S4. Global distribution of *Paragonimus westermani *and *Paragonimus skrjabini*. (a) 1951–2000. (b) 2001–2023.

Additional file 7: Fig. S5. Global distribution of *Clonorchis sinensis*, *Opisthorchis felineus*, *Opisthorchis viverrini*, and *Metorchis orientalis*. (a) 1901–1950. (b) 1951–2000. (c) 2001–2023.

Additional file 8: Fig. S6. Global distribution of *Fasciola hepatica*, *Fasciola gigantica*, and* Fasciolopsis buski*. (a) 1901–1950. (b) 1951–2000. (c) 2001–2023.

Additional file 9: Fig. S7. Global distribution of *Heterophyes heterophyes* and *Heterophyes nocens*. (a) 1901–1950. (b) 1951–2000. (c) 2001–2023.

Additional file 10: Fig. S8. Global distribution of *Haplorchis pumilio *and *Haplorchis taichui*. (a) 1901–1950. (b) 1951–2000. (c) 2001–2023.

Additional file 11: Fig. S9. Global distribution of *Metagonimus yokagawai*, *Stellantchasmus falcatus, *and *Centrocestus formosanus*. (a) 1901–1950. (b) 1951–2000. (c) 2001–2023.

Additional file 12: Fig. S10. Global distribution of *Eurytrema cladorchis*. (a) 1951–2000. (b) 2001–2023.

## Data Availability

Not applicable.

## Abbreviation

WHO: World Health Organization

## References

[1] Marsden P. Clinicai Parasitology, 9th Editon, Beaver PC, Jung RC, Cupp EW. Lea and Febiger, Philadelphia, 1984. Revista da Sociedade Brasileira de Medicina Tropical. 1984;17:219.

[2] Doughty BL, Baron S (1996). Schistosomes and Other Trematodes. Medical Microbiology.

[3] Galaktionov KV, Dobrovolskij AA. In: Fried B, Graczyk TK, editors. The Biology and Evolution of Trematodes. Springer Netherlands; 2003. https://www.researchgate.net/publication/257234158_Galaktionov_K_V_A_A_Dobrovolskij_2003_The_Biology_and_Evolution_of_Trematodes_Kluwer_Academic_Publ_Boston_Dordrecht_London.

[4] Crotti M (2013). Digenetic Trematodes: an existence as .parasites Brief general overview. Microbiol Med.

[5] Chai JY, Jung BK (2022). General overview of the current status of human foodborne trematodiasis. Parasitology.

[6] Chai JY, Jung BK (2020). Foodborne intestinal flukes: A brief review of epidemiology and geographical distribution. Acta Trop.

[7] Chai JY, Jung BK (2019). Epidemiology of trematode infections: an update. Adv Exp Med Biol.

[8] Chai JY, Jung BK (2017). Fishborne zoonotic heterophyid infections: An update. Food Waterborne Parasitol.

[9] Fürst T, Keiser J, Utzinger J (2012). Global burden of human food-borne trematodiasis: a systematic review and meta-analysis. Lancet Infect Dis.

[10] Hung NM, Madsen H, Fried B (2013). Global status of fish-borne zoonotic trematodiasis in humans. Acta Parasitol.

[11] Tidman R, Kanankege KST, Bangert M, Abela-Ridder B (2023). Global prevalence of 4 neglected foodborne trematodes targeted for control by WHO: a scoping review to highlight the gaps. PLoS Negl Trop Dis.

[12] Munn Z, Moola S, Riitano D, Lisy K (2014). The development of a critical appraisal tool for use in systematic reviews addressing questions of prevalence. Int J Health Policy Manag.

[13] Kalinda C, Mindu T, Chimbari MJ (2020). A systematic review and meta-analysis quantifying schistosomiasis infection burden in pre-school aged children (PreSAC) in sub-Saharan Africa for the period 2000–2020. PLoS One.

[14] McManus DP, Dunne DW, Sacko M, Utzinger J, Vennervald BJ, Zhou XN (2018). Schistosomiasis. Nat Rev Dis Primers.

[15] Katz N (2008). The discovery of schistosomiasis mansoni in Brazil. Acta Trop.

[16] Sinderson HC, Mills EA (1923). Rectal papillomata in *Schistosoma haematobium* infestations. Br Med J.

[17] Nishimura K, Hung T (1997). Current views on geographic distribution and modes of infection of neurohelminthic diseases. J Neurol Sci.

[18] Boissier J, Grech-Angelini S, Webster BL, Allienne JF, Huyse T, Mas-Coma S (2016). Outbreak of urogenital schistosomiasis in Corsica (France): an epidemiological case study. Lancet Infect Dis.

[19] Lingscheid T, Kurth F, Clerinx J, Marocco S, Trevino B, Schunk M (2017). Schistosomiasis in European travelers and migrants: analysis of 14 years TropNet surveillance data. Am J Trop Med Hyg.

[20] Badmos K, Komolafe A, Rotimi O (2007). Schistosomiasis presenting as acute appendicitus. East Afr Med J.

[21] De Duarte GalhardoAlbuquerque RD, Mahomoodally MF, Lobine D, Suroowan S, Rengasamy KR (2020). Botanical products in the treatment and control of schistosomiasis: Recent studies and distribution of active plant resources according to affected regions. Biology.

[22] Augusto G, Nalá R, Casmo V, Sabonete A, Mapaco L, Monteiro J (2009). Geographic distribution and prevalence of schistosomiasis and soil-transmitted helminths among schoolchildren in Mozambique. Am J Trop Med Hyg.

[23] Cutajar CL (1983). The role of schistosomiasis in urolithiasis. Br J Urol.

[24] Kurup R, Hunjan GS (2010). Epidemiology and control of Schistosomiasis and other intestinal parasitic infections among school children in three rural villages of south Saint Lucia. J Vector Borne Dis.

[25] Abebe N, Erko B, Medhin G, Berhe N (2014). Clinico-epidemiological study of schistosomiasis mansoni in Waja-Timuga, District of Alamata, northern Ethiopia. Parasit Vectors.

[26] Rujeni N, Bayingana JB, Nyandwi E, Ntakarutimana A, Kagabo J, Rutayisire R (2022). Prevalence mapping of *Schistosoma mansoni* among pre-school age children in Rwanda. Front Pediatr.

[27] Nigo MM, Odermatt P, Salieb-Beugelaar GB, Morozov O, Battegay M, Hunziker PR (2021). Epidemiology of *Schistosoma mansoni* infection in Ituri Province, north-eastern Democratic Republic of the Congo. PLoS Negl Trop Dis.

[28] Ponpetch K, Erko B, Bekana T, Richards L, Liang S (2021). Biogeographical characteristics of *Schistosoma mansoni* endemic areas in Ethiopia: a systematic review and meta analysis. Infect Dis Poverty.

[29] Ishii A, Tsuji M, Tada I (2003). History of Katayama disease: schistosomiasis japonica in Katayama district, Hiroshima, Japan. Parasitol Int.

[30] Yogore MG, Lewert RM, Blas BL (1984). Seroepidemiology of schistosomiasis japonica by ELISA in the Philippines. III. Selective mass chemotherapy with praziquantel in a control program. Am J Trop Med Hyg.

[31] Rollinson D, Knopp S, Levitz S, Stothard JR, Tchuem Tchuenté L-A, Garba A (2013). Time to set the agenda for schistosomiasis elimination. Acta Trop.

[32] Soares Magalhães RJ, Barnett AG, Clements ACA (2011). Geographical analysis of the role of water supply and sanitation in the risk of helminth infections of children in West Africa. Proc Natl Acad Sci USA.

[33] Guo SY, Li L, Zhang LJ, Li YL, Li SZ, Xu J (2021). From the One Health perspective: schistosomiasis japonica and flooding. Pathogens.

[34] Colley DG, Bustinduy AL, Secor WE, King CH (2014). Human schistosomiasis. Lancet.

[35] Voge M, Bruckner D, Bruce JI (1978). Schistosoma mekongi sp. N. from man and animals, compared with four geographic strains of Schistosoma japonicum. J Parasitol.

[36] Uthailak N, Adisakwattana P, Thiangtrongjit T, Limpanont Y, Chusongsang P, Chusongsang Y (2022). Discovery of *Schistosoma mekongi* circulating proteins and antigens in infected mouse sera. PLoS One.

[37] Ohmae H, Sinuon M, Kirinoki M, Matsumoto J, Chigusa Y, Socheat D (2004). Schistosomiasis mekongi: from discovery to control. Parasitol Int.

[38] Olveda DU, Li Y, Olveda RM, Lam AK, Chau TN, Harn DA (2013). Bilharzia: pathology, diagnosis, management and control. Trop Med Surg.

[39] Attwood SW, Fatih FA, Upatham ES (2008). DNA-sequence variation among *Schistosoma mekongi* populations and related taxa; phylogeography and the current distribution of Asian schistosomiasis. PLoS Negl Trop Dis.

[40] Anazawa K (1929). On a human case of *Echinostoma revolutum* and its infection route. Taiwan Igahkai Zasshi.

[41] Lu SC (1982). Echinostomiasis in Taiwan. Int J Zoonoses.

[42] Morgan JAT, Blair D (1998). Mitochondrial ND1 gene sequences used to identify echinostome isolates from Australia and New Zealand. Int J Parasitol.

[43] Chai JY, Cho J, Chang T, Jung BK, Sohn WM (2020). Taxonomy of *Echinostoma**revolutum* and 37-collar-spined *Echinostoma* spp.: a historical review. Korean J Parasitol.

[44] Huffman JE, Fried B (1990). *Echinostoma* and echinostomiasis. Adv Parasitol.

[45] Faltýnková A, Georgieva S, Soldánová M, Kostadinova A (2015). A re-assessment of species diversity within the “revolutum” group of *Echinostoma Rudolphi*, 1809 (Digenea: Echinostomatidae) in Europe. Syst Parasitol.

[46] Fried B, Huffman JE (1996). The biology of the intestinal trematode *Echinostoma caproni*. Adv Parasitol.

[47] Detwiler JT, Zajac AM, Minchella DJ, Belden LK (2012). Revealing cryptic parasite diversity in a definitive host: Echinostomes in Muskrats. J Parasitol.

[48] Detwiler JT, Bos DH, Minchella DJ (2010). Revealing the secret lives of cryptic species: Examining the phylogenetic relationships of echinostome parasites in North America. Mol Phylogenet Evol.

[49] Poland GA (1985). Outbreak of parasitic gastroenteritis among travelers returning from Africa. Arch Intern Med.

[50] Chai JY, Lee SH (1990). Intestinal trematodes of humans in Korea: Metagonimus, heterophyids and echinostomes. Korean J Parasitol.

[51] Toledo R, Fried B. Helminth-trematode: *Echinostoma*. Encyclopedia of Food Safety. 2014;134–9.

[52] Mao SP (1991). Protozoan and helminth parasites of humans in mainland China. Int J Parasitol.

[53] Chai JY, Lee SH (2002). Food-borne intestinal trematode infections in the Republic of Korea. Parasitol Int.

[54] Dimitrov V, Kanev I, Bezprozvanich V, Radev V (1998). Argentophilic structures of the miracidium of *Echinochasmus perfoliatus* (Trematoda: Echinosmatidae). Parasite.

[55] Chai JY, Park JH, Jung BK, Guk SM, Kim JL, Shin EH (2009). Echinostome Infections in the striped-field Mouse, *Apodemus agrarius*, and the Ussuri white-toothed shrew, *Crocidura lasiura*, Caught near the demilitarized Zone, Gyeonggi-do (Province), Republic of Korea. Korean J Parasitol.

[56] Awad-Alla ME, Abdien HMF, Dessouki AA (2010). Prevalence of bacteria and parasites in White Ibis in Egypt. Vet Ital.

[57] Al-Sabi M, Halasa T, Kapel C (2014). Infections with cardiopulmonary and intestinal helminths and sarcoptic mange in red foxes from two different localities in Denmark. Acta Parasitol.

[58] Doanh PN, Shinohara A, Horii Y, Habe S, Nawa Y (2009). Discovery of *Paragonimus**westermani* in Vietnam and its molecular phylogenetic status in P. *westermani* complex. Parasitol Res.

[59] Kerbert C (1878). Zur Trematoden-kenntnis. ZoolAnzeiger.

[60] Iwagami M, Ho LY, Su K, Lai PF, Fukushima M, Nakano M (2000). Molecular phylogeographic studies on *Paragonimus westermani* in Asia. J Helminthol.

[61] Kim DC (1984). Paragonimus westermani: life cycle, intermediate hosts, transmission to man and geographical distribution in Korea. Arzneimittelforschung.

[62] Devi KR, Narain K, Mahanta J, Nirmolia T, Blair D, Saikia SP (2013). Presence of three distinct genotypes within the *Paragonimus westermani* complex in northeastern India. Parasitology.

[63] Kuntz RE (1969). Biology of *Paragonimus westermani* (Kerbert, 1878) Braun, 1899: infection in the crab host (Eriocheir japonicus de Haan) on Taiwan. Trans Am Microsc Soc.

[64] Miyazaki I, Terasaki K, Iwata K (1978). Natural infection of muscle of wild boars in Japan by immature *Paragonimus**westermani* (Kerbert 1878). J Parasitol.

[65] Miyazaki I, Kawashima K, Tan MH (1968). *Parathelphusa**maculata* de Man, 1879, a New Crustacean host record for *Paragonimus**westermani* (Kerbert, 1878) in Malaysia. J Parasitol.

[66] Keiser J, Utzinger J (2005). Emerging foodborne trematodiasis. Emerg Infect Dis.

[67] Chen HT. The occurrence of a new type of *Paragonimus* and some clinical problems related to lung flukes in China. Annual Report 1958, Chung Shan Medical College, Guangzhou. 1959. p. 192–3. (in Chinese). https://link.springer.com/article/10.1007/s11230-004-1378-5.

[68] Zhou XJ, Yang Q, Tan QH, Zhang LY, Shi LB, Zou JX (2021). *Paragonimus* and its hosts in China: an update. Acta Trop.

[69] Su TC (1983). Occurrence of *Pagumogonimus**skrjabini* in Fangshan county, Hubei province. Chin J Parasitol Parasit Dis.

[70] Xiao JH, Chen CE, Zhang WC, Nie CX, Li BW (1993). Comparison of the repetitive DNA sequences between *Paragonimus**westermani* and *Pagumogonimus**skrjabini* from six areas. Chin J Parasitol Parasit Dis.

[71] Yang JS, Chen M, Feng Z, Blair D (2000). *Paragonimus* and paragonimiasis in China: a review of the literature. Chin J Parasitol Parasit Dis.

[72] Watson FC (1918). *Clonorchis**sinensis* infection of the gall-bladder and biliary passages. Ann Surg.

[73] Mas-Coma S, Bargues M (1997). Human liver flukes: a review. Res Rev Parasitol.

[74] Miyazaki I. An illustrated book of helminthic zoonoses. An illustrated book of helminthic zoonoses. 1991; Available from: https://www.cabdirect.org/cabdirect/abstract/19922089143. Cited 2023 May 17.

[75] Slepchenko S (2020). *Opisthorchis**felineus* as the basis for the reconstruction of migrations using archaeoparasitological materials. J Archaeol Sci.

[76] Wykoff DE, Harinasuta C, Juttijudata P, Winn MM (1965). *Opisthorchis**viverrini* in thailand–the life cycle and comparison with *O*. *felineus*. J Parasitol.

[77] Tanabe H (1920). Ein neuer Metorchis aus der Galleblase der Hausente. Acta Scholae Med Univ Imp Kioto.

[78] Lin J, Chen Y, Li Y (2001). The discovery of natural infection of human with *Metorchis**orientalis* and the investigation of its focus. Chin J Zoonoses.

[79] Cheng Y, Xu L, Chen B, Li LS, Zhang RY, Lin CX (2005). Survey on the current status of important human parasitic infections in Fujian Province. Chin J Parasitol Parasit Dis.

[80] Yamaguti S (1958). Systema Helminthum. Vol 1 The Digenetic Trematodes of Vertebrates- Part 1.

[81] Zhan X, Li C, Wu H, Sun E, Zhu Y (2017). Investigation on the endemic characteristics of *Metorchis**orientalis* in Huainan area. China Nutr Hosp.

[82] Mas-Coma S, Valero MA, Bargues MD (2022). Human and animal fascioliasis: origins and worldwide evolving Scenario. Clin Microbiol Rev.

[83] Mas-Coma S, Valero MA, Bargues MD (2009). Chapter 2. Fasciola, lymnaeids and human fascioliasis, with a global overview on disease transmission, epidemiology, evolutionary genetics, molecular epidemiology and control. Adv Parasitol.

[84] Mas-Coma S (2005). Epidemiology of fascioliasis in human endemic areas. J Helminthol.

[85] Sadykov VM. Detection of Fasciola in deceased persons in Samarkand Province. Med Parazitol (Mosk). 1988;(4):71–3. (in Russian). https://pubmed.ncbi.nlm.nih.gov/2973553/. 2973553

[86] Gonzalez C, Valero MA, Curtale F, Montresor A, Mas-Coma S, Abdel-Wahab Y (2003). Hyperendemic fascioliasis associated with schistosomiasis in villages in the Nile Delta of Egypt. Am J Trop Med Hyg.

[87] Boray JC (1969). Experimental Fascioliasis in Australia. Adv Parasitol.

[88] Mas-Coma S, Bargues MD, Valero MA (2005). Fascioliasis and other plant-borne trematode zoonoses. Int J Parasitol.

[89] Kang BK, Jung BK, Lee YS, Hwang IK, Lim H, Cho J (2014). A case of *Fasciola**hepatica* infection mimicking cholangiocarcinoma and ITS-1 sequencing of the worm. Korean J Parasitol.

[90] Cobbold TS. Description of a new trematode worm (*Fasciola gigantica*). The Edinburgh New Philosophical Journal, New Series. 1855:262–7. https://www.gbif.org/species/2505829.

[91] Spithill TW, Smooker PM, Bruce D (1999). *Fasciola**gigantica*: epidemiology, control, immunology and molecular biology. Fasciolosis.

[92] Tum S, Puotinen ML, Skerratt LF, Chan B, Sothoeun S (2007). Validation of a geographic information system model for mapping the risk of fasciolosis in cattle and buffaloes in Cambodia. Vet Parasitol.

[93] Quang TD, Duong TH, Richard-Lenoble D, Odermatt P, Khammanivong K (2008). Emergence in humans of fascioliasis (from *Fasciola**gigantica*) and intestinal distomatosis (from *Fasciolopsis buski*) in Laos. Sante.

[94] Liu GH, Gasser RB, Young ND, Song HQ, Ai L, Zhu XQ (2014). Complete mitochondrial genomes of the ‘intermediate form’ of *Fasciola* and *Fasciola**gigantica*, and their comparison with *F*. *hepatica*. Parasit Vectors.

[95] Han H, Peng J, Hong Y, Zhang M, Han Y, Liu D (2013). MicroRNA expression profile in different tissues of BALB/c mice in the early phase of *Schistosoma**japonicum* infection. Mol Biochem Parasitol.

[96] Cook GC (1996). George Busk, FRS (1807–1886): surgeon, zoologist, parasitologist and palaeontologist. Trans R Soc Trop Med Hyg.

[97] Wu X, Wang W, Li Q, Xue Q, Li Y, Li S (2020). Case Report: Surgical intervention for *Fasciolopsis buski* infection: a literature review. Am J Trop Med Hyg.

[98] Pv M, Pm S (1972). Epidemiological study of fasciolopsis buski in Palghar Taluk. Indian J Public Health.

[99] Fiamma M, Longoni SS, Ngo TMC, Le Phan MT, Santona A, Ton Nu PA (2015). Fasciolopsis buski infection in a Vietnamese pregnant woman with systemic lupus erythematosus. J Infect Dev Ctries.

[100] Rohela M, Jamaiah I, Menon J, Rachel J (2005). Fasciolopsiasis: a first case report from Malaysia. Southeast Asian J Trop Med Public Health.

[101] Jha AK, Jha SK (2020). Endoscopic diagnosis of *Fasciolopsis**buski*: Revisited (with video). JGH Open.

[102] Kumari N, Kumar M, Rai A, Acharya A (2006). Intestinal trematode infection in North Bihar. JNMA J Nepal Med Assoc.

[103] Saikia D, Prasad YK, Dahal S, Ghatani S (2022). *Fasciolopsis buski* detected in humans in Bihar and Pigs in Assam. India.

[104] Medical Research in Egypt (1935). Nature.

[105] Chai JY, Seo BS, Lee SH, Hong SJ, Sohn WM (1986). Human infections by *Heterophyes**heterophyes* and H. dispar imported from Saudi Arabia. Korean J Parasitol.

[106] Eom KS (1985). Heterophyid trematodes (*Heterophyes**heterophyes* and H. *dispar*) human infections imported from Sudan to Korea. Korean J Parasitol.

[107] Kagei N, Hayashi S, Kato K (1980). On the Heterophyid trematoda (*Heterophyes**heterophyes*) infection cases imported from Egypt to Japan. Japan J Trop Med Hyg.

[108] Elmonir W, Elaadli H, Amer A, El-Sharkawy H, Bessat M, Mahmoud SF (2021). Prevalence of intestinal parasitic infections and their associated risk factors among preschool and school children in Egypt. PLoS One.

[109] Onji Y, Nishio T (1916). On the trematodes whose intermediate host is brackish water fishes. Chiba Igaku Semmon Gakko Zasshi.

[110] Seo BS, Hong ST, Chai JY (1981). Studies on intestinal trematodes in Korea. III. Natural human infections of *Pygidiopsis**summa* and *Heterophyes**heterophyes**nocens*. Seoul J Med.

[111] Yu SH, Mott KE. Epidemiology and morbidity of food-borne intestinal trematode infections. Trop Dis Bull. 1994;91:R125–52.

[112] Namchote S, Sritongtae S, Butnin S, Chai JY, Jung BK, Sohn WM (2015). Larval stage of trematodes obtained from brackish water snails in the central and east coast of the gulf of Thailand. Sci Res Essays.

[113] Looss A (1899). Weitere Beiträge zur Kenntniss der Trematoden-Fauna Aegyptens, zugleich Versuch einer natürlichen Gliederung des Genus Distomum Retzius. Zoologische Jahrbücher.

[114] Radomyos P, Bunnag D, Harinasuta T (1983). Haplorchis pumilio (Looss) infection in man in northeastern Thailand. Southeast Asian J Trop Med Public Health.

[115] Nishigori M (1924). The life cycles of two new species of Heterophyidae, Monorchotrema *taihokui* and *M*. *taichui*, found in Formosa. Preliminary note. Taiwan Igakkwai Zasshi, Taihoku.

[116] Beaver PC, Jung RC, Cupp EW. Clin Parasitol. 1984. https://www.researchgate.net/publication/262510185_Clinicai_Parasitology_9th_Editon_Beaver_PC_Jung_R_C_Cupp_E_W_Lea_and_Febiger_Philadelphia_1984.

[117] Chai JY, Shin EH, Lee SH, Rim HJ (2009). Foodborne intestinal flukes in Southeast Asia. Korean J Parasitol.

[118] Belizario VY, de Leon WU, Bersabe MJJ, Purnomo null, Baird JK, Bangs MJ. A focus of human infection by *Haplorchis taichui* (Trematoda: Heterophyidae) in the southern Philippines. J Parasitol. 2004;90:1165–9. 10.1645/GE-3304RN 15562620

[119] Soh CT, Ahn YK (1978). Epidemiological study on *Metagonimus**yokogawai* infection along Boseong River In Jeonra Nam Do. Korea Korean J Parasitol.

[120] Shimazu T, Kino H (2015). *Metagonimus**yokogawai* (Trematoda: Heterophyidae): From discovery to designation of a neotype. Korean J Parasitol.

[121] Chai JY, Han ET, Park YK, Guk SM, Kim JL, Lee SH (2000). High endemicity of *Metagonimus yokogawai* infection among residents of Samchok-shi. Kangwon-do Korean J Parasitol.

[122] Kino H, Oishi H, Ohno Y, Ishiguro M (2002). An endemic human infection with *Heterophyes**nocens**onji**et**nishio* 1916 at Mikkabi-cho, Shizuoka. Japan Japan J Trop Med Hyg.

[123] WoRMS - World Register of Marine Species - Cornatrium Onji & Nishio, 1916. https://marinespecies.org/aphia.php?p=taxdetails&id=725686. Accessed 18 May 2023

[124] Alicata JE (1938). A case of intestinal heterophyidiasis of man in Hawaii. JAMA.

[125] Thien PC, Dalsgaard A, Thanh BN, Olsen A, Murrell KD (2007). Prevalence of fishborne zoonotic parasites in important cultured fish species in the Mekong Delta. Vietnam Parasitol Res.

[126] Nishigori M. On a New Species of Fluke, *Stamnosoma formosanum*, and its Life-History. Journal of the Medical Association of Formosa; Available from: https://www.semanticscholar.org/paper/On-a-New-Species-of-Fluke%2C-Stamnosoma-formosanum%2C-Nishigori/08208046de91144f217dc221050475a8baefd89b. Cited 2023 May 18

[127] Yu S, Xu L, Jiang Z, Xu S, Han J, Zhu Y (1994). Report on the first nationwide survey of the distribution of human parasites in China. 1. Regional distribution of parasite species. Chin J Parasitol Parasit Dis.

[128] Srisawangwong T, Sithithaworn P, Tesana S (1997). Metacercariae isolated from cyprinoid fishes in Khon Kaen District by digestion technic. Southeast Asian J Trop Med Public Health.

[129] Bayssade-Dufour C, Albaret JL, Ow-Yang CK (1982). Sensillae and protonephridia of *cercaria* of *Centrocestus**formosanus* and *Centrocestus* sp. (Centrocestinae, Heterophyidae). Ann Parasitol Hum Comp.

[130] Ortega C, Fajardo R, Enríquez R (2009). Trematode *Centrocestus**formosanus* infection and distribution in ornamental fishes in Mexico. J Aquat Anim Health.

[131] Chai JY, Sohn WM, Yong TS, Eom KS, Min DY, Lee MY (2013). *Centrocestus**formosanus* (Heterophyidae): Human Infections and the Infection Source in Lao PDR. J Parasitol.

[132] De NV, Le TH (2011). Human infections of fish-borne trematodes in Vietnam: Prevalence and molecular specific identification at an endemic commune in Nam Dinh province. Exp Parasitol.

[133] Patarwut L, Chontananarth T, Chai JY, Purivirojkul W (2020). Infections of digenetic trematode metacercariae in wrestling halfbeak, *Dermogenys**pusilla* from Bangkok Metropolitan Region in Thailand. Korean J Parasitol.

[134] Jones A (1985). *Eurytrema**cladorchis* Chin, Li & Wei, 1965 (Trematoda: Dicrocoeliidae), a little known species from China and Nepal. Syst Parasitol.

[135] Thang TN, Thuy PD, Lan NTK, Doanh PN, Duyen DTH, Ichikawa-Seki M (1907). Morphological and molecular characterization of *Eurytrema* spp. Looss, 1907 detected in domestic water buffaloes and cattle in northern Vietnam. J Vet Med Sci.

[136] Mohanta U, Ichikawa-Seki M, Hayashi K, Itagaki T (2015). Morphological and molecular characterization of *Eurytrema cladorchis* parasitizing cattle (*Bos indicus*) in Bangladesh. Parasitol Res.

[137] Hanafiah M, Helmi TZ, Sutriana A, Bahi M (2021). Morphology and molecular identification of Eurytrema spp. worm in Aceh cattle, Indonesia. Biodiversitas (Surak).

[138] World Health Organization. Available from: https://www.who.int. Cited 2023 May 16

[139] Zhou XN, Bergquist R, Leonardo L, Yang GJ, Yang K, Sudomo M (2010). Schistosomiasis japonica control and research needs. Adv Parasitol.

[140] Khieu V, Sayasone S, Muth S, Kirinoki M, Laymanivong S, Ohmae H (2019). Elimination of Schistosomiasis mekongi from endemic areas in Cambodia and the Lao People’s Democratic Republic: current status and plans. Trop Med Infect Dis.

[141] Toledo R, Esteban JG (2016). An update on human echinostomiasis. Trans R Soc Trop Med Hyg.

[142] Sayasone S, Tesana S, Utzinger J, Hatz C, Akkhavong K, Odermatt P (2009). Rare human infection with the trematode *Echinochasmus japonicus* in Lao PDR. Parasitol Int.

[143] El-Azazy OME, Abdou NEMI, Khalil AI, Al-Batel MK, Majeed QAH, Henedi AAR (2015). Potential zoonotic trematodes recovered in stray cats from Kuwait Municipality, Kuwait. Korean J Parasitol.

[144] Shin SS, Oh DS, Ahn KS, Cho SH, Lee WJ, Na BK (2015). Zoonotic intestinal trematodes in stray cats (*Felis catus*) from riverside areas of the Republic of Korea. Korean J Parasitol.

[145] Keiser J, Utzinger J (2009). Food-borne trematodiases. Clin Microbiol Rev.

[146] Pakharukova MY, Mordvinov VA (2016). The liver fluke *Opisthorchis**felineus*: biology, epidemiology and carcinogenic potential. Trans R Soc Trop Med Hyg.

[147] Webb CM, Cabada MM (2018). Recent developments in the epidemiology, diagnosis, and treatment of *Fasciola* infection. Curr Opin Infect Dis.

[148] Liu D (2013). Molecular Detection of Human Parasitic Pathogens, CRC Press.

[149] Chai JY, Park JH, Han ET, Shin EH, Kim JL, Guk SM (2004). Prevalence of *Heterophyes**nocens* and P*ygydiopsis**summa* infections among residents of the western and southern coastal islands of the Republic of Korea. Am J Trop Med Hyg.

[150] Kim TS, Cho SH, Huh S, Kong Y, Sohn WM, Hwang SS (2009). A nationwide survey on the prevalence of intestinal parasitic infections in the Republic of Korea, 2004. Korean J Parasito.

[151] Cho SH, Cho PY, Lee DM, Kim TS, Kim IS, Hwang EJ (2010). Epidemiological survey on the infection of intestinal flukes in residents of Muan-gun, Jeollanam-do, the Republic of Korea. Korean J Parasitol.

[152] Maguire JH, Bennett JE, Dolin R, Blaser MJ (2015). 290 - Trematodes (Schistosomes and Liver, Intestinal, and Lung Flukes). Mandell, Douglas, and Bennett’s Principles and Practice of Infectious Diseases.

[153] Chongti T, Tongmin L (1980). Investigations on eurytremosis of cattle and goats in mountainous regions of north Fujian. Acta Zool Sin.

[154] Deol AK, Fleming FM, Calvo-Urbano B, Walker M, Bucumi V, Gnandou I (2019). Schistosomiasis - Assessing progress toward the 2020 and 2025 global goals. N Engl J Med.

[155] Esteban JG, Muñoz-Antoli C, Toledo R, Fried B (2009). Echinostomes: systematics and life cycles. The Biology of Echinostomes.

[156] Liang YS, Coles GC, Dai JR, Zhu YC, Doenhoff MJ (2002). Adult worm tegumental damage and egg-granulomas in praziquantel-resistant and -susceptible *Schistosoma mansoni* treated in vivo. J Helminthol.

[157] Wang W, Wang L, Liang YS (2012). Susceptibility or resistance of praziquantel in human schistosomiasis: a review. Parasitol Res.

[158] Alonso D, Muñoz J, Gascón J, Valls ME, Corachan M (2006). Failure of standard treatment with praziquantel in two returned travelers with *Schistosoma haematobium* infection. Am J Trop Med Hyg.

