# Supplementary material for: Global distribution of zoonotic digenetic trematodes: a scoping review
Source: Infect Dis Poverty. 2024 Jun 14;13:46. doi: 10.1186/s40249-024-01208-1 (PMC11177464; doi:10.1186/s40249-024-01208-1)
Supplement: Supplementary file 1 — Additional file 1: Search strategies in each database. [file 40249_2024_1208_MOESM1_ESM.docx]

**Additional file 1.** Search strategies in each database.

| **a. PubMed Search Query**  **“Schistosoma haematobium” AND (“Asia” OR “Africa” OR “America” OR “Antarctica” OR “Europe” OR “Oceania” OR “China”) AND (“human” OR “animal”)**  **“Schistosoma mansoni” AND (“Asia” OR “Africa” OR “America” OR “Antarctica” OR “Europe” OR “Oceania” OR “China”) AND (“human” OR “animal”)**  **“Schistosoma japonicum” AND (“Asia” OR “Africa” OR “America” OR “Antarctica” OR “Europe” OR “Oceania” OR “China”) AND (“human” OR “animal”)**  **“Schistosoma mekongi” AND (“Asia” OR “Africa” OR “America” OR “Antarctica” OR “Europe” OR “Oceania” OR “China”) AND (“human” OR “animal”)**  **“Echinostoma revolutum” AND (“Asia” OR “Africa” OR “America” OR “Antarctica” OR “Europe” OR “Oceania” OR “China”) AND (“human” OR “animal”)**  **“Isthmiophora hortensis” AND (“Asia” OR “Africa” OR “America” OR “Antarctica” OR “Europe” OR “Oceania” OR “China”) AND (“human” OR “animal”)**  **“Echinochasmus japonicus” AND (“Asia” OR “Africa” OR “America” OR “Antarctica” OR “Europe” OR “Oceania” OR “China”) AND (“human” OR “animal”)**  **“Echinochasmus perfoliatus” AND (“Asia” OR “Africa” OR “America” OR “Antarctica” OR “Europe” OR “Oceania” OR “China”) AND (“human” OR “animal”)**  **“Paragonimus westermani” AND (“Asia” OR “Africa” OR “America” OR “Antarctica” OR “Europe” OR “Oceania” OR “China”) AND (“human” OR “animal”)**  **“Paragonimus skrjabini” AND (“Asia” OR “Africa” OR “America” OR “Antarctica” OR “Europe” OR “Oceania” OR “China”) AND (“human” OR “animal”)**  **“Clonorchis sinensis” AND (“Asia” OR “Africa” OR “America” OR “Antarctica” OR “Europe” OR “Oceania” OR “China”) AND (“human” OR “animal”)**  **“Opisthorchis felineus” AND (“Asia” OR “Africa” OR “America” OR “Antarctica” OR “Europe” OR “Oceania” OR “China”) AND (“human” OR “animal”)**  **“Opisthorchis viverrini” AND (“Asia” OR “Africa” OR “America” OR “Antarctica” OR “Europe” OR “Oceania” OR “China”) AND (“human” OR “animal”)**  **“Metorchis orientalis” AND (“Asia” OR “Africa” OR “America” OR “Antarctica” OR “Europe” OR “Oceania” OR “China”) AND (“human” OR “animal”)**  **“Fasciola hepatica” AND (“Asia” OR “Africa” OR “America” OR “Antarctica” OR “Europe” OR “Oceania” OR “China”) AND (“human” OR “animal”)**  **“Fasciola gigantica” AND (“Asia” OR “Africa” OR “America” OR “Antarctica” OR “Europe” OR “Oceania” OR “China”) AND (“human” OR “animal”)**  **“Fasciolopsis buski” AND (“Asia” OR “Africa” OR “America” OR “Antarctica” OR “Europe” OR “Oceania” OR “China”) AND (“human” OR “animal”)**  **“Heterophyes heterophyes” AND (“Asia” OR “Africa” OR “America” OR “Antarctica” OR “Europe” OR “Oceania” OR “China”) AND (“human” OR “animal”)**  **“Heterophyes nocens” AND (“Asia” OR “Africa” OR “America” OR “Antarctica” OR “Europe” OR “Oceania” OR “China”) AND (“human” OR “animal”)**  **“Haplorchis pumilio” AND (“Asia” OR “Africa” OR “America” OR “Antarctica” OR “Europe” OR “Oceania” OR “China”) AND (“human” OR “animal”)**  **“Haplorchis taichui” AND (“Asia” OR “Africa” OR “America” OR “Antarctica” OR “Europe” OR “Oceania” OR “China”) AND (“human” OR “animal”)**  **“Metagonimus yokagawai” AND (“Asia” OR “Africa” OR “America” OR “Antarctica” OR “Europe” OR “Oceania” OR “China”) AND (“human” OR “animal”)**  **“Stellantchasmus falcatus” AND (“Asia” OR “Africa” OR “America” OR “Antarctica” OR “Europe” OR “Oceania” OR “China”) AND (“human” OR “animal”)**  **“Centrocestus formosanus” AND (“Asia” OR “Africa” OR “America” OR “Antarctica” OR “Europe” OR “Oceania” OR “China”) AND (“human” OR “animal”)**  **“Eurytrema cladorchis” AND (“Asia” OR “Africa” OR “America” OR “Antarctica” OR “Europe” OR “Oceania” OR “China”) AND (“human” OR “animal”)** |
| --- |
| **b. Web of Science Search Query**  **“Schistosoma haematobium” AND (“Asia” OR “Africa” OR “America” OR “Antarctica” OR “Europe” OR “Oceania” OR “China”) AND (“human” OR “animal”) AND "distribution"**  **“Schistosoma mansoni” AND (“Asia” OR “Africa” OR “America” OR “Antarctica” OR “Europe” OR “Oceania” OR “China”) AND (“human” OR “animal”) AND "distribution"**  **“Schistosoma japonicum” AND (“Asia” OR “****Africa” OR “America” OR “Antarctica” OR “Europe” OR “Oceania” OR “China”) AND (“human” OR “animal”) AND "distribution"**  **“Schistosoma mekongi” AND (“Asia” OR “Africa” OR “America” OR “Antarctica” OR “Europe” OR “Oceania” OR “China”) AND (“human” OR “animal”) AND "distribution"**  **“Echinostoma revolutum” AND (“Asia” OR “Africa” OR “America” OR “Antarctica” OR “Europe” OR “Oceania” OR “China”) AND (“human” OR “animal”) AND "distribution"**  **“Isthmiophora hortensis” AND (“Asia” OR “Africa” OR “America” OR “Antarctica” OR “Europe” OR “Oceania” OR “China”) AND (“human” OR “animal”) AND "distribution"**  **“Echinochasmus japonicus” AND (“Asia” OR “Africa” OR “America” OR “Antarctica” OR “Europe” OR “Oceania” OR “China”) AND (“human” OR “animal”) AND "distribution"**  **“Echinochasmus perfoliatus” AND (“Asia” OR “Africa” OR “America” OR “Antarctica” OR “Europe” OR “Oceania” OR “China”) AND (“human” OR “animal”) AND "distribution"**  **“Paragonimus westermani” AND (“Asia” OR “Africa” OR “America” OR “Antarctica” OR “Europe” OR “Oceania” OR “China”) AND (“human” OR “animal”) AND "distribution"**  **“Paragonimus skrjabini” AND (“Asia” OR “Africa” OR “America” OR “Antarctica” OR “Europe” OR “Oceania” OR “China”) AND (“human” OR “animal”) AND "distribution"**  **“Clonorchis sinensis” AND (“Asia” OR “Africa” OR “America” OR “Antarctica” OR “Europe” OR “Oceania” OR “China”) AND (“human” OR “animal”) AND "distribution"**  **“Opisthorchis felineus” AND (“Asia” OR “Africa” OR “America” OR “Antarctica” OR “Europe” OR “Oceania” OR “China”) AND (“human” OR “animal”) AND "distribution"**  **“Opisthorchis viverrini” AND (“Asia” OR “Africa” OR “America” OR “Antarctica” OR “Europe” OR “Oceania” OR “China”) AND (“human” OR “animal”) AND "distribution"**  **“Metorchis orientalis” AND (“Asia” OR “Africa” OR “America” OR “Antarctica” OR “Europe” OR “Oceania” OR “China”) AND (“human” OR “animal”) AND "distribution"**  **“Fasciola hepatica” AND (“Asia” OR “Africa” OR “America” OR “Antarctica” OR “Europe” OR “Oceania” OR “China”) AND (“human” OR “animal”) AND "distribution"**  **“Fasciola gigantica” AND (“Asia” OR “Africa” OR “America” OR “Antarctica” OR “Europe” OR “Oceania” OR “China”) AND (“human” OR “animal”) AND "distribution"**  **“Fasciolopsis buski” AND (“Asia” OR “Africa” OR “America” OR “Antarctica” OR “Europe” OR “Oceania” OR “China”) AND (“human” OR “animal”) AND "distribution"**  **“Heterophyes heterophyes” AND (“Asia” OR “Africa” OR “America” OR “Antarctica” OR “Europe” OR “Oceania” OR “China”) AND (“human” OR “animal”) AND "distribution"**  **“Heterophyes nocens” AND (“Asia” OR “Africa” OR “America” OR “Antarctica” OR “Europe” OR “Oceania” OR “China”) AND (“human” OR “animal”) AND "distribution"**  **“Haplorchis pumilio” AND (“Asia” OR “Africa” OR “America” OR “Antarctica” OR “Europe” OR “Oceania” OR “China”) AND (“human” OR “animal”) AND "distribution"**  **“Haplorchis taichui” AND (“Asia” OR “Africa” OR “America” OR “Antarctica” OR “Europe” OR “Oceania” OR “China”) AND (“human” OR “animal”) AND "distribution"**  **“Metagonimus yokagawai” AND (“Asia” OR “Africa” OR “America” OR “Antarctica” OR “Europe” OR “Oceania” OR “China”) AND (“human” OR “animal”) AND "distribution"**  **“Stellantchasmus falcatus” AND (“Asia” OR “Africa” OR “America” OR “Antarctica” OR “Europe” OR “Oceania” OR “China”) AND (“human” OR “animal”) AND "distribution"**  **“Centrocestus formosanus” AND (“Asia” OR “Africa” OR “America” OR “Antarctica” OR “Europe” OR “Oceania” OR “China”) AND (“human” OR “animal”) AND "distribution"**  **“Eurytrema cladorchis” AND (“Asia” OR “Africa” OR “America” OR “Antarctica” OR “Europe” OR “Oceania” OR “China”) AND (“human” OR “animal”) AND "distribution"** |
| **c. Google Scholar Search Query**  **allintitle: Asia OR Africa OR America OR Antarctica OR Europe OR Oceania OR China "Schistosoma haematobium"**  **allintitle: Asia OR Africa OR America OR Antarctica OR Europe OR Oceania OR China "Schistosoma mansoni"**  **allintitle: Asia OR Africa OR America OR Antarctica OR Europe OR Oceania OR China "Schistosoma japonicum"**  **allintitle: Asia OR Africa OR America OR Antarctica OR Europe OR Oceania OR China "Schistosoma mekongi"**  **allintitle: Asia OR Africa OR America OR Antarctica OR Europe OR Oceania OR China "Echinostoma revolutum"**  **allintitle: Asia OR Africa OR America OR Antarctica OR Europe OR Oceania OR China "Isthmiophora hortensis"**  **allintitle: Asia OR Africa OR America OR Antarctica OR Europe OR Oceania OR China “Echinochasmus japonicus”**  **allintitle: Asia OR Africa OR America OR Antarctica OR Europe OR Oceania OR China “Echinochasmus perfoliatus”**  **allintitle: Asia OR Africa OR America OR Antarctica OR Europe OR Oceania OR China “Paragonimus westermani”**  **allintitle: Asia OR Africa OR America OR Antarctica OR Europe OR Oceania OR China “Paragonimus skrjabini”**  **allintitle: Asia OR Africa OR America OR Antarctica OR Europe OR Oceania OR China “Clonorchis sinensis”**  **allintitle: Asia OR Africa OR America OR Antarctica OR Europe OR Oceania OR China “Opisthorchis felineus”**  **allintitle: Asia OR Africa OR America OR Antarctica OR Europe OR Oceania OR China “Opisthorchis viverrini”**  **allintitle: Asia OR Africa OR America OR Antarctica OR Europe OR Oceania OR China “Metorchis orientalis”**  **allintitle: Asia OR Africa OR America OR Antarctica OR Europe OR Oceania OR China “Fasciola hepatica”**  **allintitle: Asia OR Africa OR America OR Antarctica OR Europe OR Oceania OR China “Fasciola gigantica”**  **allintitle: Asia OR Africa OR America OR Antarctica OR Europe OR Oceania OR China “Fasciolopsis buski”**  **allintitle: Asia OR Africa OR America OR Antarctica OR Europe OR Oceania OR China “Heterophyes heterophyes”**  **allintitle: Asia OR Africa OR America OR Antarctica OR Europe OR Oceania OR China “Heterophyes nocens”**  **allintitle: Asia OR Africa OR America OR Antarctica OR Europe OR Oceania OR China “Haplorchis pumilio”**  **allintitle: Asia OR Africa OR America OR Antarctica OR Europe OR Oceania OR China “Haplorchis taichui”**  **allintitle: Asia OR Africa OR America OR Antarctica OR Europe OR Oceania OR China “Metagonimus yokagawai”**  **allintitle: Asia OR Africa OR America OR Antarctica OR Europe OR Oceania OR China “Stellantchasmus falcatus”**  **allintitle: Asia OR Africa OR America OR Antarctica OR Europe OR Oceania OR China “Centrocestus formosanus”**  **allintitle: Asia OR Africa OR America OR Antarctica OR Europe OR Oceania OR China “Eurytrema cladorchis”** |
| **d. CNKI Search Query**  **Schistosoma haematobium * (Asia + Africa + America + Antarctica + Europe + Oceania + China) * (human + animal)**  **Schistosoma mansoni * (Asia + Africa + America + Antarctica + Europe + Oceania + China) * (human + animal)**  **Schistosoma japonicum * (Asia + Africa + America + Antarctica + Europe + Oceania + China) * (human + animal)**  **Schistosoma mekongi * (Asia + Africa + America + Antarctica + Europe + Oceania + China) * (human + animal)**  **Echinostoma revolutum * (Asia + Africa + America + Antarctica + Europe + Oceania + China) * (human + animal)**  **Isthmiophora hortensis * (Asia + Africa + America + Antarctica + Europe + Oceania + China) * (human + animal)**  **Echinochasmus japonicus * (Asia + Africa + America + Antarctica + Europe + Oceania + China) * (human + animal)**  **Echinochasmus perfoliatus * (Asia + Africa + America + Antarctica + Europe + Oceania + China) * (human + animal)**  **Paragonimus westermani * (Asia + Africa + America + Antarctica + Europe + Oceania + China) * (human + animal)**  **Paragonimus skrjabini * (Asia + Africa + America + Antarctica + Europe + Oceania + China) * (human + animal)**  **Clonorchis sinensis * (Asia + Africa + America + Antarctica + Europe + Oceania + China) * (human + animal)**  **Opisthorchis felineus * (Asia + Africa + America + Antarctica + Europe + Oceania + China) * (human + animal)**  **Opisthorchis viverrini * (Asia + Africa + America + Antarctica + Europe + Oceania + China) * (human + animal)**  **Metorchis orientalis * (Asia + Africa + America + Antarctica + Europe + Oceania + China) * (human + animal)**  **Fasciola hepatica * (Asia + Africa + America + Antarctica + Europe + Oceania + China) * (human + animal)**  **Fasciola gigantica * (Asia + Africa + America + Antarctica + Europe + Oceania + China) * (human + animal)**  **Fasciolopsis buski * (Asia + Africa + America + Antarctica + Europe + Oceania + China) * (human + animal)**  **Heterophyes heterophyes * (Asia + Africa + America + Antarctica + Europe + Oceania + China) * (human + animal)**  **Heterophyes nocens * (Asia + Africa + America + Antarctica + Europe + Oceania + China) * (human + animal)**  **Haplorchis pumilio * (Asia + Africa + America + Antarctica + Europe + Oceania + China) * (human + animal)**  **Haplorchis taichui * (Asia + Africa + America + Antarctica + Europe + Oceania + China) * (human + animal)**  **Metagonimus yokagawai * (Asia + Africa + America + Antarctica + Europe + Oceania + China) * (human + animal)**  **Stellantchasmus falcatus * (Asia + Africa + America + Antarctica + Europe + Oceania + China) * (human + animal)**  **Centrocestus formosanus * (Asia + Africa + America + Antarctica + Europe + Oceania + China) * (human + animal)**  **Eurytrema cladorchis * (Asia + Africa + America + Antarctica + Europe + Oceania + China) * (human + animal)** |
| **e. Wanfang Search Query**  **“Schistosoma haematobium” AND (“Asia” OR “Africa” OR “America” OR “Antarctica” OR “Europe” OR “Oceania” OR “China”) AND (“human” OR “animal”)**  **“Schistosoma mansoni” AND (“Asia” OR “Africa” OR “America” OR “Antarctica” OR “Europe” OR “Oceania” OR “China”) AND (“human” OR “animal”)**  **“Schistosoma japonicum” AND (“Asia” OR “Africa” OR “America” OR “Antarctica” OR “Europe” OR “Oceania” OR “China”) AND (“human” OR “animal”)**  **“Schistosoma mekongi” AND (“Asia” OR “Africa” OR “America” OR “Antarctica” OR “Europe” OR “Oceania” OR “China”) AND (“human” OR “animal”)**  **“Echinostoma revolutum” AND (“Asia” OR “Africa” OR “America” OR “Antarctica” OR “Europe” OR “Oceania” OR “China”) AND (“human” OR “animal”)**  **“Isthmiophora hortensis” AND (“Asia” OR “Africa” OR “America” OR “Antarctica” OR “Europe” OR “Oceania” OR “China”) AND (“human” OR “animal”)**  **“Echinochasmus japonicus” AND (“Asia” OR “Africa” OR “America” OR “Antarctica” OR “Europe” OR “Oceania” OR “China”) AND (“human” OR “animal”)**  **“Echinochasmus perfoliatus” AND (“Asia” OR “Africa” OR “America” OR “Antarctica” OR “Europe” OR “Oceania” OR “China”) AND (“human” OR “animal”)**  **“Paragonimus westermani” AND (“Asia” OR “Africa” OR “America” OR “Antarctica” OR “Europe” OR “Oceania” OR “China”) AND (“human” OR “animal”)**  **“Paragonimus skrjabini” AND (“Asia” OR “Africa” OR “America” OR “Antarctica” OR “Europe” OR “Oceania” OR “China”) AND (“human” OR “animal”)**  **“Clonorchis sinensis” AND (“Asia” OR “Africa” OR “America” OR “Antarctica” OR “Europe” OR “Oceania” OR “China”) AND (“human” OR “animal”)**  **“Opisthorchis felineus” AND (“Asia” OR “Africa” OR “America” OR “Antarctica” OR “Europe” OR “Oceania” OR “China”) AND (“human” OR “animal”)**  **“Opisthorchis viverrini” AND (“Asia” OR “Africa” OR “America” OR “Antarctica” OR “Europe” OR “Oceania” OR “China”) AND (“human” OR “animal”)**  **“Metorchis orientalis” AND (“Asia” OR “Africa” OR “America” OR “Antarctica” OR “Europe” OR “Oceania” OR “China”) AND (“human” OR “animal”)**  **“Fasciola hepatica” AND (“Asia” OR “Africa” OR “America” OR “Antarctica” OR “Europe” OR “Oceania” OR “China”) AND (“human” OR “animal”)**  **“Fasciola gigantica” AND (“Asia” OR “Africa” OR “America” OR “Antarctica” OR “Europe” OR “Oceania” OR “China”) AND (“human” OR “animal”)**  **“Fasciolopsis buski” AND (“Asia” OR “Africa” OR “America” OR “Antarctica” OR “Europe” OR “Oceania” OR “China”) AND (“human” OR “animal”)**  **“Heterophyes heterophyes” AND (“Asia” OR “Africa” OR “America” OR “Antarctica” OR “Europe” OR “Oceania” OR “China”) AND (“human” OR “animal”)**  **“Heterophyes nocens” AND (“Asia” OR “Africa” OR “America” OR “Antarctica” OR “Europe” OR “Oceania” OR “China”) AND (“human” OR “animal”)**  **“Haplorchis pumilio” AND (“Asia” OR “Africa” OR “America” OR “Antarctica” OR “Europe” OR “Oceania” OR “China”) AND (“human” OR “animal”)**  **“Haplorchis taichui” AND (“Asia” OR “Africa” OR “America” OR “Antarctica” OR “Europe” OR “Oceania” OR “China”) AND (“human” OR “animal”)**  **“Metagonimus yokagawai” AND (“Asia” OR “Africa” OR “America” OR “Antarctica” OR “Europe” OR “Oceania” OR “China”) AND (“human” OR “animal”)**  **“Stellantchasmus falcatus” AND (“Asia” OR “Africa” OR “America” OR “Antarctica” OR “Europe” OR “Oceania” OR “China”) AND (“human” OR “animal”)**  **“Centrocestus formosanus” AND (“Asia” OR “Africa” OR “America” OR “Antarctica” OR “Europe” OR “Oceania” OR “China”) AND (“human” OR “animal”)**  **“Eurytrema cladorchis” AND (“Asia” OR “Africa” OR “America” OR “Antarctica” OR “Europe” OR “Oceania” OR “China”) AND (“human” OR “animal”)** |
