# Supplementary material for: Global distribution of zoonotic digenetic trematodes: a scoping review
Source: Infect Dis Poverty. 2024 Jun 14;13:46. doi: 10.1186/s40249-024-01208-1 (PMC11177464; doi:10.1186/s40249-024-01208-1)
Supplement: Supplementary file 2 — Additional file 2: Quality assessment report of included articles in this review. [file 40249_2024_1208_MOESM2_ESM.docx]

**Additional file 2.** Quality assessment report of included articles in this review.

The quality of the included articles was assessed using the Joanna Briggs Institute (JBI) Prevalence Critical Appraisal Tool [1]. The tool assessed each article on the following:

1. Sample representative of the target population
2. Study participants recruited in an appropriate way
3. Sample size adequate
4. Study subjects and setting described in detail
5. Data analysis was conducted with enough coverage of the identified sample
6. Validity of methods used to identify the condition of interest
7. Condition measured in a standard, reliable way for all participants
8. Appropriate statistical analysis
9. Adequacy of response rate; were low response rates managed?
10. Sub-population of interest identified using objective criteria

| Author(s) and Year | Was the sample representative of the target population? | Were study participants recruited in an appropriate way? | Was the sample size adequate? | Were the study subjects and setting described in detail? | Was data analysis conducted with sufficient coverage of the identified sample? | Were valid methods used for the identification of the condition? | Was the condition measured in a standard, reliable way for all participants? | Was there appropriate statistical analysis? | Was the response rate adequate, and if not, was the low response rate managed appropriately? | Were target subpopulations identified using objective criteria? | Quality score |
| --- | --- | --- | --- | --- | --- | --- | --- | --- | --- | --- | --- |
| Chai J-Y et al., 2022 | Yes | Yes | Yes | Yes | Not specified | Yes | Not specified | Yes | NA | Not specified | 6 |
| Chai J-Y et al., 2020 | Yes | Yes | Yes | Yes | Not specified | Yes | Not specified | Yes | NA | Not specified | 6 |
| Chai J-Y et al., 2019 | Yes | Yes | Yes | Yes | Not specified | Yes | Not specified | Yes | NA | Not specified | 6 |
| Chai J-Y et al., 2017 | Yes | Yes | Yes | Yes | Not specified | Yes | Not specified | Yes | NA | Not specified | 6 |
| Fürst T et al., 2012 | Yes | Yes | Yes | Yes | Not specified | Yes | Not specified | Yes | NA | Not specified | 6 |
| Hung NM et al., 2013 | Yes | Yes | Yes | Yes | Not specified | Yes | Not specified | Yes | NA | Not specified | 6 |
| Tidman R et al., 2023 | Yes | Yes | Yes | Yes | Not specified | Yes | Not specified | Yes | NA | Not specified | 6 |
| Munn Z et al., 2014 | Yes | Yes | Yes | Yes | Not specified | Yes | Not specified | Yes | NA | Not specified | 6 |
| Kalinda C et al., 2020 | Yes | Yes | Yes | Yes | Not specified | Yes | Not specified | Yes | NA | Not specified | 6 |
| McManus DP et al., 2018 | Yes | Yes | Yes | Yes | Not specified | Yes | Not specified | Yes | NA | Not specified | 6 |
| Katz N et al., 2008 | Yes | Yes | Yes | Yes | Not specified | Yes | Not specified | Yes | NA | Not specified | 6 |
| Sinderson HC et al., 1923 | Yes | Yes | No | Yes | Not specified | Yes | Not specified | No | NA | Not specified | 4 |
| Nishimura K et al., 1997 | Yes | Yes | Yes | Yes | Not specified | Yes | Not specified | Yes | NA | Not specified | 6 |
| Boissier J et al., 2016 | Yes | Yes | Yes | Yes | Not specified | Yes | Not specified | Yes | NA | Not specified | 6 |
| Lingscheid T et al., 2017 | Yes | Yes | Yes | Yes | Not specified | Yes | Not specified | Yes | NA | Not specified | 6 |
| Badmos K et al., 2007 | Yes | Yes | Yes | Yes | Not specified | Yes | Not specified | Yes | NA | Not specified | 6 |
| Duarte Galhardo De Albuquerque RD et al., 2020 | Yes | Yes | Yes | Yes | Not specified | Yes | Not specified | Yes | NA | Not specified | 6 |
| Augusto G et al., 2009 | Yes | Yes | Yes | Yes | Not specified | Yes | Not specified | Yes | NA | Not specified | 6 |
| Cutajar CL et al., 1983 | Yes | Yes | Yes | Yes | Not specified | Yes | Not specified | Yes | NA | Not specified | 6 |
| Kurup R et al.,2010 | Yes | Yes | Yes | Yes | Not specified | Yes | Not specified | Yes | NA | Not specified | 6 |
| Abebe N et al., 2014 | Yes | Yes | Yes | Yes | Not specified | Yes | Not specified | Yes | NA | Not specified | 6 |
| Rujeni N et al., 2022 | Yes | Yes | Yes | Yes | Not specified | Yes | Not specified | Yes | NA | Not specified | 6 |
| Mm N et al., 2021 | Yes | Yes | Yes | Yes | Not specified | Yes | Not specified | Yes | NA | Not specified | 6 |
| Ponpetch K et al., 2021 | Yes | Yes | Yes | Yes | Not specified | Yes | Not specified | Yes | NA | Not specified | 6 |
| Ishii A et al., 2003 | Yes | Yes | Yes | Yes | Not specified | Yes | Not specified | Yes | NA | Not specified | 6 |
| Yogore MG et al., 1984 | Yes | Yes | Yes | Yes | Not specified | Yes | Not specified | Yes | NA | Not specified | 6 |
| Rollinson D et al., 2013 | Yes | Yes | Yes | Yes | Not specified | Yes | Not specified | Yes | NA | Not specified | 6 |
| Soares Magalhães RJ et al., 2011 | Yes | Yes | Yes | Yes | Not specified | Yes | Not specified | Yes | NA | Not specified | 6 |
| Guo S-Y et al., 2021 | Yes | Yes | Yes | Yes | Not specified | Yes | Not specified | Yes | NA | Not specified | 6 |
| Colley DG et al., 2014 | Yes | Yes | Yes | Yes | Not specified | Yes | Not specified | Yes | NA | Not specified | 6 |
| Anazawa K et al.,1929 | Yes | Yes | No | Yes | Not specified | Yes | Not specified | No | NA | Not specified | 4 |
| Lu SC et al., 1982 | Yes | Yes | Yes | Yes | Not specified | Yes | Not specified | Yes | NA | Not specified | 6 |
| Morgan JAT et al., 1998 | Yes | Yes | Yes | Yes | Not specified | Yes | Not specified | Yes | NA | Not specified | 6 |
| Chai J-Y et al., 2020 | Yes | Yes | Yes | Yes | Not specified | Yes | Not specified | Yes | NA | Not specified | 6 |
| Huffman JE et al., 1990 | Yes | Yes | Yes | Yes | Not specified | Yes | Not specified | Yes | NA | Not specified | 6 |
| Faltýnková A et al., 2015 | Yes | Yes | Yes | Yes | Not specified | Yes | Not specified | Yes | NA | Not specified | 6 |
| Fried B et al., 1996 | Yes | Yes | Yes | Yes | Not specified | Yes | Not specified | Yes | NA | Not specified | 6 |
| Detwiler JT et al., 2012 | Yes | Yes | Yes | Yes | Not specified | Yes | Not specified | Yes | NA | Not specified | 6 |
| Detwiler JT et al., 2010 | Yes | Yes | Yes | Yes | Not specified | Yes | Not specified | Yes | NA | Not specified | 6 |
| Poland GA et al., 1985 | Yes | Yes | Yes | Yes | Not specified | Yes | Not specified | Yes | NA | Not specified | 6 |
| Chai JY et al., 1990 | Yes | Yes | Yes | Yes | Not specified | Yes | Not specified | Yes | NA | Not specified | 6 |
| Toledo R et al., 2014 | Yes | Yes | Yes | Yes | Not specified | Yes | Not specified | Yes | NA | Not specified | 6 |
| Toledo R et al., 2016 | Yes | Yes | Yes | Yes | Not specified | Yes | Not specified | Yes | NA | Not specified | 6 |
| Mao SP et al., 1991 | Yes | Yes | Yes | Yes | Not specified | Yes | Not specified | Yes | NA | Not specified | 6 |
| Chai J-Y et al., 2002 | Yes | Yes | Yes | Yes | Not specified | Yes | Not specified | Yes | NA | Not specified | 6 |
| Dimitrov V et al., 1998 | Yes | Yes | Yes | Yes | Not specified | Yes | Not specified | Yes | NA | Not specified | 6 |
| Chai J-Y et al., 2009 | Yes | Yes | Yes | Yes | Not specified | Yes | Not specified | Yes | NA | Not specified | 6 |
| Awad-Alla ME et al., 2010 | Yes | Yes | Yes | Yes | Not specified | Yes | Not specified | Yes | NA | Not specified | 6 |
| Al-Sabi M et al., 2014 | Yes | Yes | Yes | Yes | Not specified | Yes | Not specified | Yes | NA | Not specified | 6 |
| Sayasone S et al., 2009 | Yes | Yes | No | Yes | Not specified | Yes | Not specified | Yes | NA | Not specified | 5 |
| El-Azazy OME et al., 2015 | Yes | Yes | Yes | Yes | Not specified | Yes | Not specified | Yes | NA | Not specified | 6 |
| Shin S-S et al., 2015 | Yes | Yes | Yes | Yes | Not specified | Yes | Not specified | Yes | NA | Not specified | 6 |
| Doanh PN et al., 2009 | Yes | Yes | Yes | Yes | Not specified | Yes | Not specified | Yes | NA | Not specified | 6 |
| Kerbert et al., 1878 | Yes | Yes | No | Yes | Not specified | Yes | Not specified | No | NA | Not specified | 4 |
| Iwagami M et al., 2000 | Yes | Yes | Yes | Yes | Not specified | Yes | Not specified | Yes | NA | Not specified | 6 |
| Kim DC et al., 1984 | Yes | Yes | Yes | Yes | Not specified | Yes | Not specified | Yes | NA | Not specified | 6 |
| Devi KR et al., 2013 | Yes | Yes | Yes | Yes | Not specified | Yes | Not specified | Yes | NA | Not specified | 6 |
| Kuntz RE et al., 1969 | Yes | Yes | Yes | Yes | Not specified | Yes | Not specified | Yes | NA | Not specified | 6 |
| Miyazaki I et al., 1978 | Yes | Yes | Yes | Yes | Not specified | Yes | Not specified | Yes | NA | Not specified | 6 |
| Miyazaki I et al., 1968 | Yes | Yes | No | Yes | Not specified | Yes | Not specified | Yes | NA | Not specified | 5 |
| Keiser J et al., 2005 | Yes | Yes | Yes | Yes | Not specified | Yes | Not specified | Yes | NA | Not specified | 6 |
| Chen H.T. et al., 1958 | Yes | Yes | No | Yes | Not specified | Yes | Not specified | No | NA | Not specified | 4 |
| Zhou X-J et al., 2021 | Yes | Yes | Yes | Yes | Not specified | Yes | Not specified | Yes | NA | Not specified | 6 |
| Su TC et al., 1983 | Yes | Yes | Yes | Yes | Not specified | Yes | Not specified | Yes | NA | Not specified | 6 |
| Xiao JH et al., 1993 | Yes | Yes | Yes | Yes | Not specified | Yes | Not specified | Yes | NA | Not specified | 6 |
| Yang JS et al., 2000 | Yes | Yes | Yes | Yes | Not specified | Yes | Not specified | Yes | NA | Not specified | 6 |
| Watson FC et al., 1918 | Yes | Yes | No | Yes | Not specified | Yes | Not specified | No | NA | Not specified | 4 |
| Mas-Coma S et al., 1997 | Yes | Yes | Yes | Yes | Not specified | Yes | Not specified | Yes | NA | Not specified | 6 |
| Slepchenko S et al., 2020 | Yes | Yes | Yes | Yes | Not specified | Yes | Not specified | Yes | NA | Not specified | 6 |
| Wykoff DE et al., 1965 | Yes | Yes | Yes | Yes | Not specified | Yes | Not specified | Yes | NA | Not specified | 6 |
| Keiser J et al., 2009 | Yes | Yes | Yes | Yes | Not specified | Yes | Not specified | Yes | NA | Not specified | 6 |
| Pakharukova MY et al., 2016 | Yes | Yes | Yes | Yes | Not specified | Yes | Not specified | Yes | NA | Not specified | 6 |
| Mas-Coma S et al., 2022 | Yes | Yes | Yes | Yes | Not specified | Yes | Not specified | Yes | NA | Not specified | 6 |
| Mas‐Coma S et al., 2009 | Yes | Yes | Yes | Yes | Not specified | Yes | Not specified | Yes | NA | Not specified | 6 |
| Mas-Coma S et al., 2005 | Yes | Yes | Yes | Yes | Not specified | Yes | Not specified | Yes | NA | Not specified | 6 |
| Sadykov VM et al., 1988 | Yes | Yes | Yes | Yes | Not specified | Yes | Not specified | Yes | NA | Not specified | 6 |
| Gonzalez C et al., 2003 | Yes | Yes | Yes | Yes | Not specified | Yes | Not specified | Yes | NA | Not specified | 6 |
| Boray JC et al., 1969 | Yes | Yes | Yes | Yes | Not specified | Yes | Not specified | Yes | NA | Not specified | 6 |
| Mas-Coma S et al., 2005 | Yes | Yes | Yes | Yes | Not specified | Yes | Not specified | Yes | NA | Not specified | 6 |
| Kang BK et al., 2014 | Yes | Yes | No | Yes | Not specified | Yes | Not specified | No | NA | Not specified | 4 |
| Han H et al., 2013 | Yes | Yes | Yes | Yes | Not specified | Yes | Not specified | Yes | NA | Not specified | 6 |
| Cook GC et al., 1996 | Yes | Yes | Yes | Yes | Not specified | Yes | Not specified | Yes | NA | Not specified | 6 |
| Pv M et al., 1972 | Yes | Yes | Yes | Yes | Not specified | Yes | Not specified | Yes | NA | Not specified | 6 |
| Wu X et al., 2020 | Yes | Yes | Yes | Yes | Not specified | Yes | Not specified | Yes | NA | Not specified | 6 |
| Fiamma M et al., 2015 | Yes | Yes | No | Yes | Not specified | Yes | Not specified | No | NA | Not specified | 4 |
| Rohela M et al., 2005 | Yes | Yes | No | Yes | Not specified | Yes | Not specified | No | NA | Not specified | 4 |
| Jha AK et al., 2020 | Yes | Yes | Yes | Yes | Not specified | Yes | Not specified | Yes | NA | Not specified | 6 |
| Kumari N et al., 2006 | Yes | Yes | No | Yes | Not specified | Yes | Not specified | No | NA | Not specified | 4 |
| Saikia D et al., 2022 | Yes | Yes | Yes | Yes | Not specified | Yes | Not specified | Yes | NA | Not specified | 6 |
| Webb CM et al., 2018 | Yes | Yes | Yes | Yes | Not specified | Yes | Not specified | Yes | NA | Not specified | 6 |
| Sorvillo FJ et al., 2008 | Yes | Yes | Yes | Yes | Not specified | Yes | Not specified | Yes | NA | Not specified | 6 |
| Chai JY et al., 1986 | Yes | Yes | Yes | Yes | Not specified | Yes | Not specified | Yes | NA | Not specified | 6 |
| Eom et al., 1985 | Yes | Yes | Yes | Yes | Not specified | Yes | Not specified | Yes | NA | Not specified | 6 |
| Chai JY et al., 1992 | Yes | Yes | Yes | Yes | Not specified | Yes | Not specified | Yes | NA | Not specified | 6 |
| Elmonir W et al., 2021 | Yes | Yes | Yes | Yes | Not specified | Yes | Not specified | Yes | NA | Not specified | 6 |
| Soh CT et al., 1978 | Yes | Yes | Yes | Yes | Not specified | Yes | Not specified | Yes | NA | Not specified | 6 |
| Shimazu T et al., 2015 | Yes | Yes | Yes | Yes | Not specified | Yes | Not specified | Yes | NA | Not specified | 6 |
| Chai JY et al., 2000 | Yes | Yes | Yes | Yes | Not specified | Yes | Not specified | Yes | NA | Not specified | 6 |
| Kino H et al., 2002 | Yes | Yes | Yes | Yes | Not specified | Yes | Not specified | Yes | NA | Not specified | 6 |
| Looss A et al., 1899 | Yes | Yes | Yes | Yes | Not specified | Yes | Not specified | Yes | NA | Not specified | 6 |
| Kostadinova A et al., 2009 | Yes | Yes | Yes | Yes | Not specified | Yes | Not specified | Yes | NA | Not specified | 6 |
| Chai J-Y et al., 2009 | Yes | Yes | Yes | Yes | Not specified | Yes | Not specified | Yes | NA | Not specified | 6 |
| Radomyos P et al., 1983 | Yes | Yes | Yes | Yes | Not specified | Yes | Not specified | Yes | NA | Not specified | 6 |
| J. Alicata et al., 1938 | Yes | Yes | No | Yes | Not specified | Yes | Not specified | No | NA | Not specified | 4 |
| Dung DT et al., 2013 | Yes | Yes | Yes | Yes | Not specified | Yes | Not specified | Yes | NA | Not specified | 6 |
| Chai J-Y et al., 2016 | Yes | Yes | Yes | Yes | Not specified | Yes | Not specified | Yes | NA | Not specified | 6 |
| Cho S-H et al., 2010 | Yes | Yes | Yes | Yes | Not specified | Yes | Not specified | Yes | NA | Not specified | 6 |
| Ortega C et al., 2009 | Yes | Yes | Yes | Yes | Not specified | Yes | Not specified | Yes | NA | Not specified | 6 |
| Nishigori M et al., 1924 | Yes | Yes | Yes | Yes | Not specified | Yes | Not specified | Yes | NA | Not specified | 6 |
| Yu S et al., 1994 | Yes | Yes | Yes | Yes | Not specified | Yes | Not specified | Yes | NA | Not specified | 6 |
| Srisawangwong T et al., 1997 | Yes | Yes | Yes | Yes | Not specified | Yes | Not specified | Yes | NA | Not specified | 6 |
| Bayssade-Dufour C et al., 1982 | Yes | Yes | Yes | Yes | Not specified | Yes | Not specified | Yes | NA | Not specified | 6 |
| Ortega C et al., 2009 | Yes | Yes | Yes | Yes | Not specified | Yes | Not specified | Yes | NA | Not specified | 6 |
| Chai J-Y et al., 2013 | Yes | Yes | Yes | Yes | Not specified | Yes | Not specified | Yes | NA | Not specified | 6 |
| De NV et al., 2011 | Yes | Yes | Yes | Yes | Not specified | Yes | Not specified | Yes | NA | Not specified | 6 |
| Patarwut L et al., 2020 | Yes | Yes | Yes | Yes | Not specified | Yes | Not specified | Yes | NA | Not specified | 6 |
| Fleming BP et al., 2011 | Yes | Yes | Yes | Yes | Not specified | Yes | Not specified | Yes | NA | Not specified | 6 |
| Elmonir W et al., 2021 | Yes | Yes | Yes | Yes | Not specified | Yes | Not specified | Yes | NA | Not specified | 6 |

**Reference**

1. Munn Z, Moola S, Riitano D, Lisy K. The development of a critical appraisal tool for use in systematic reviews addressing questions of prevalence. Int J Health Policy Manag. 2014;3(3):123-8.
