# Supplementary figures and images for: Global distribution of zoonotic digenetic trematodes: a scoping review
Source: Infect Dis Poverty. 2024 Jun 14;13:46. doi: 10.1186/s40249-024-01208-1 (PMC11177464; doi:10.1186/s40249-024-01208-1)

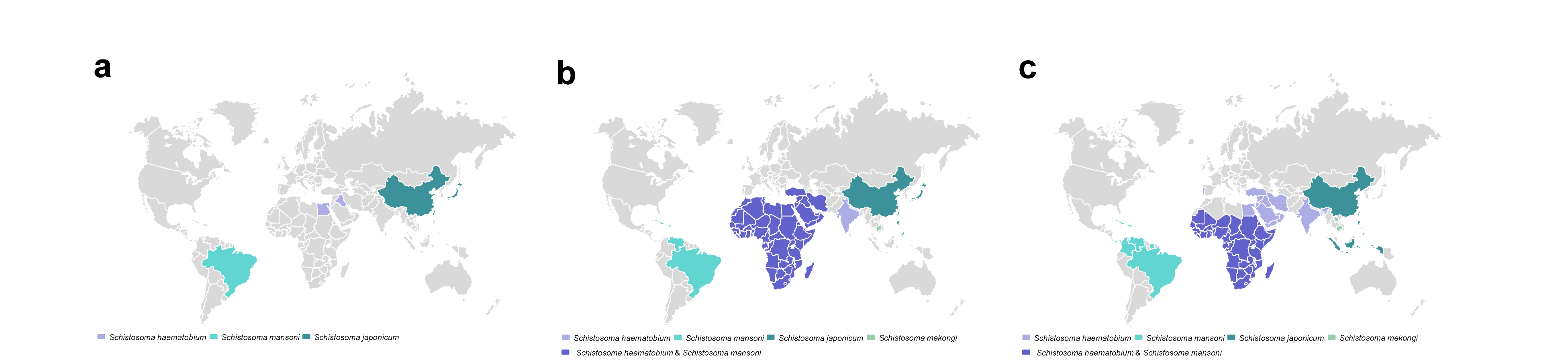

Supplement: Supplementary file 3 — Additional file 3: Fig. S1. Global distribution of Schistosoma haematobium, Schistosoma mansoni, Schistosoma japonicum, and Schistosoma mekongi. (a) 1901–1950. (b) 1951–2000. (c) 2001–2023. [file 40249_2024_1208_MOESM3_ESM.tif]

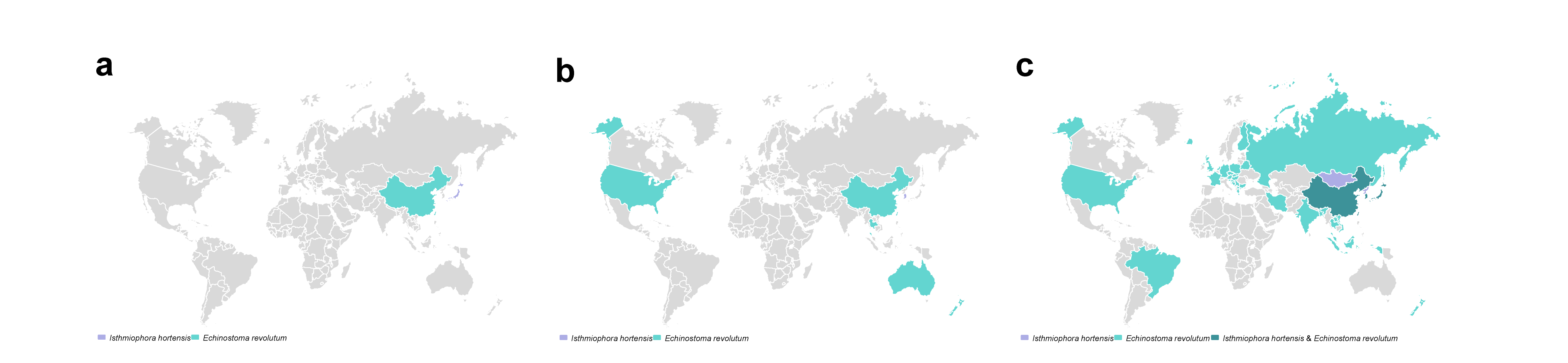

Supplement: Supplementary file 4 — Additional file 4: Fig. S2. Global distribution of Echinostoma revolutum and Isthmiophora hortensis. (a) 1901–1950. (b) 1951–2000. (c) 2001–2023. [file 40249_2024_1208_MOESM4_ESM.tif]

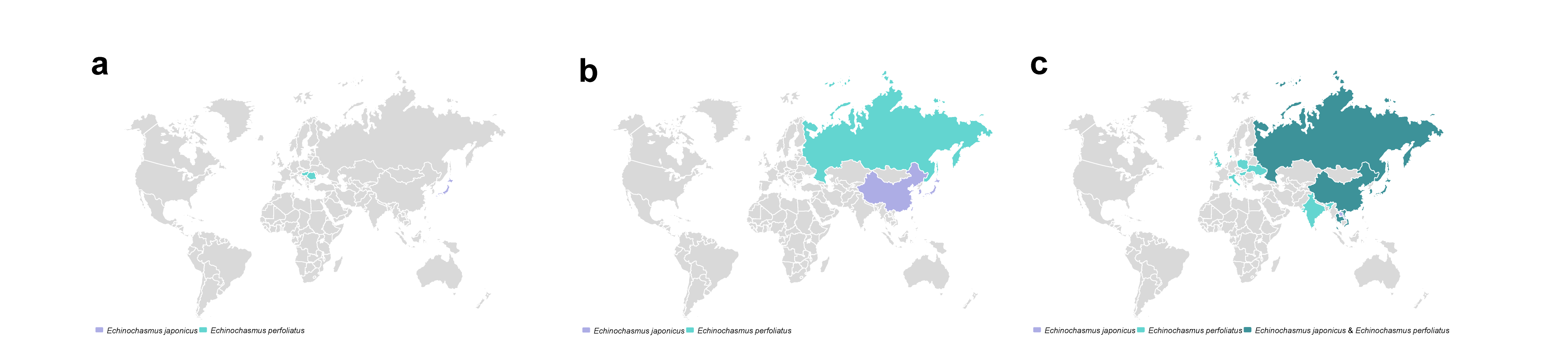

Supplement: Supplementary file 5 — Additional file 5: Fig. S3. Global distribution of Echinochasmus japonicus and Echinochasmus perfoliatus. (a) 1901–1950. (b) 1951–2000. (c) 2001–2023. [file 40249_2024_1208_MOESM5_ESM.tif]

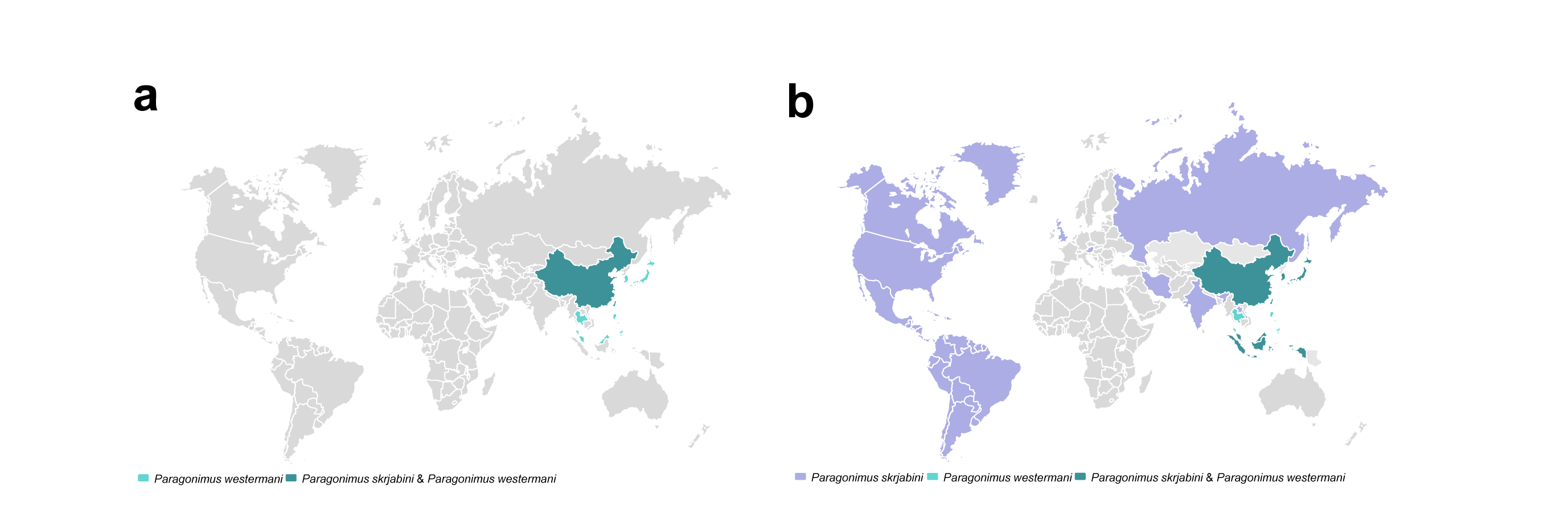

Supplement: Supplementary file 6 — Additional file 6: Fig. S4. Global distribution of Paragonimus westermani and Paragonimus skrjabini. (a) 1951–2000. (b) 2001–2023. [file 40249_2024_1208_MOESM6_ESM.tif]

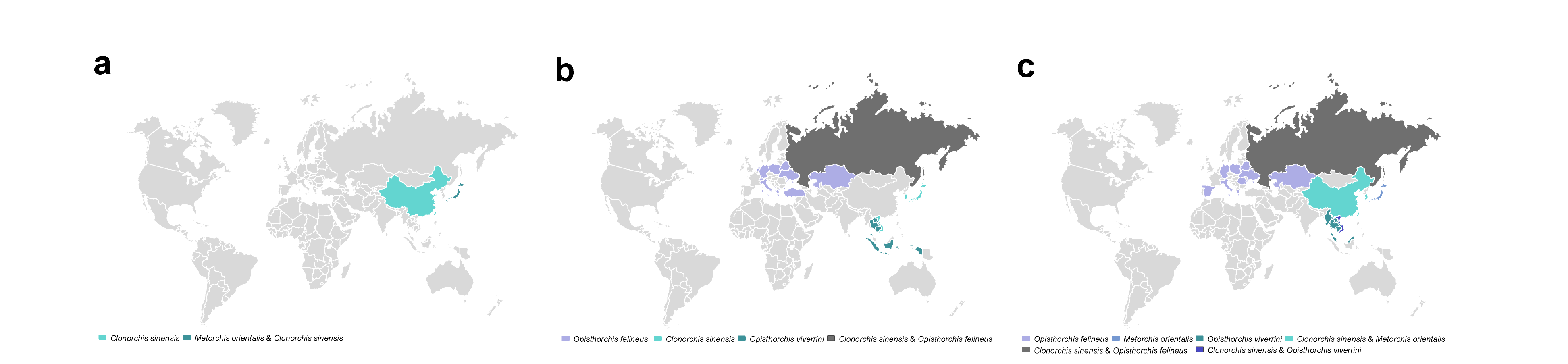

Supplement: Supplementary file 7 — Additional file 7: Fig. S5. Global distribution of Clonorchis sinensis, Opisthorchis felineus, Opisthorchis viverrini, and Metorchis orientalis. (a) 1901–1950. (b) 1951–2000. (c) 2001–2023. [file 40249_2024_1208_MOESM7_ESM.tif]

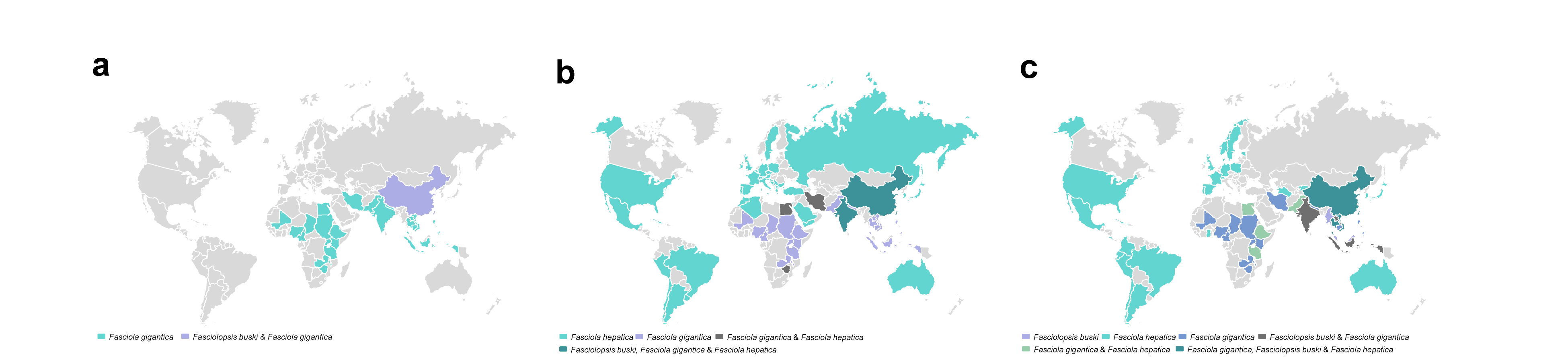

Supplement: Supplementary file 8 — Additional file 8: Fig. S6. Global distribution of Fasciola hepatica, Fasciola gigantica, and Fasciolopsis buski. (a) 1901–1950. (b) 1951–2000. (c) 2001–2023. [file 40249_2024_1208_MOESM8_ESM.tif]

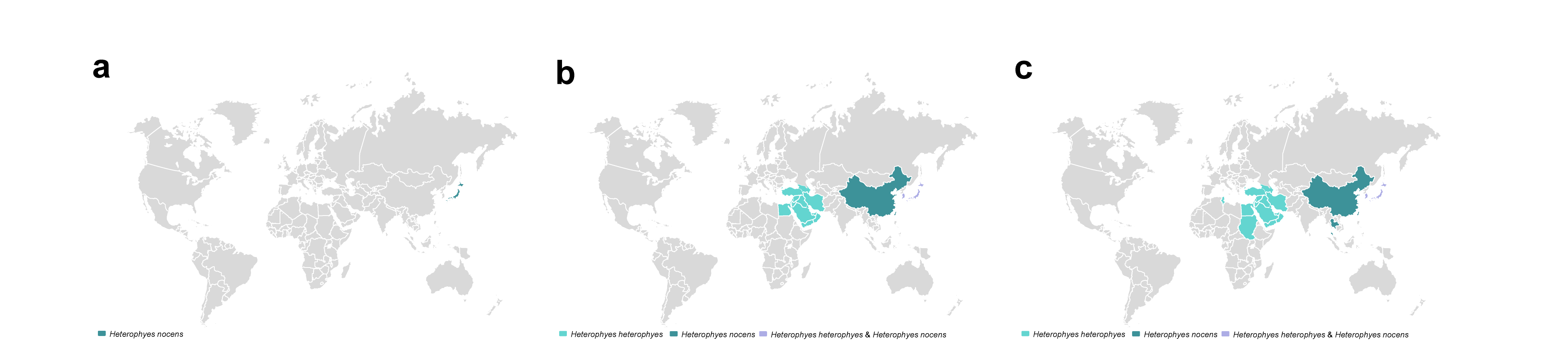

Supplement: Supplementary file 9 — Additional file 9: Fig. S7. Global distribution of Heterophyes heterophyes and Heterophyes nocens. (a) 1901–1950. (b) 1951–2000. (c) 2001–2023. [file 40249_2024_1208_MOESM9_ESM.tif]

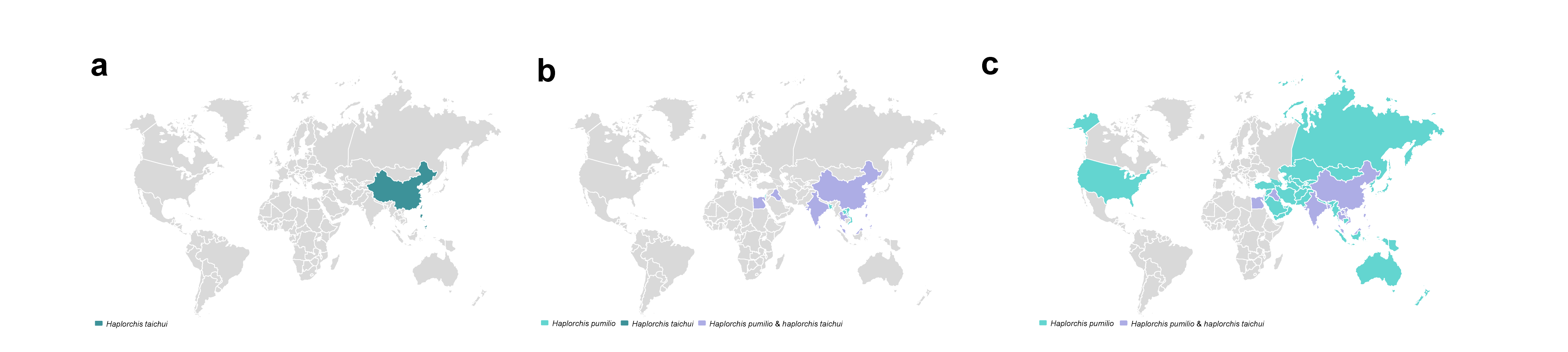

Supplement: Supplementary file 10 — Additional file 10: Fig. S8. Global distribution of Haplorchis pumilio and Haplorchis taichui. (a) 1901–1950. (b) 1951–2000. (c) 2001–2023. [file 40249_2024_1208_MOESM10_ESM.tif]

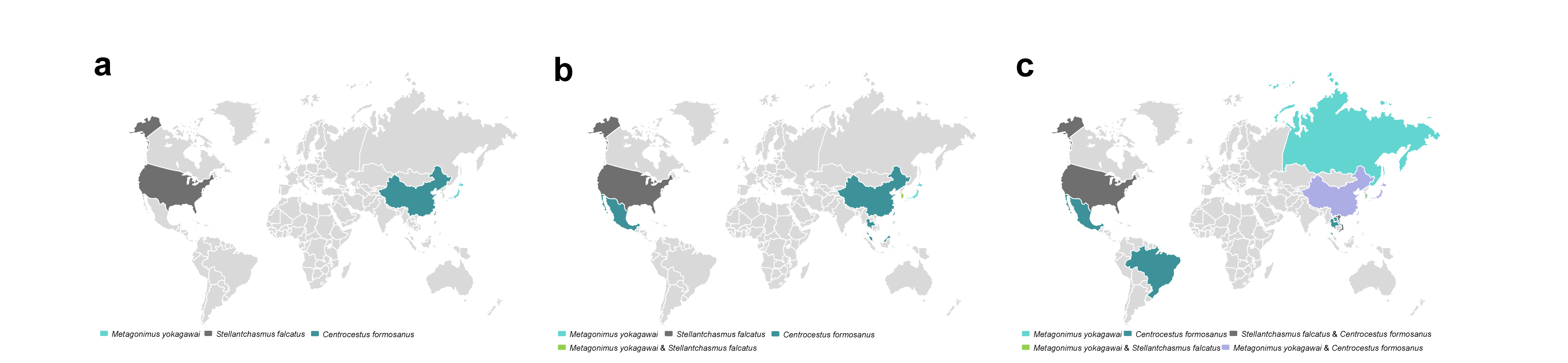

Supplement: Supplementary file 11 — Additional file 11: Fig. S9. Global distribution of Metagonimus yokagawai, Stellantchasmus falcatus, and Centrocestus formosanus. (a) 1901–1950. (b) 1951–2000. (c) 2001–2023. [file 40249_2024_1208_MOESM11_ESM.tif]

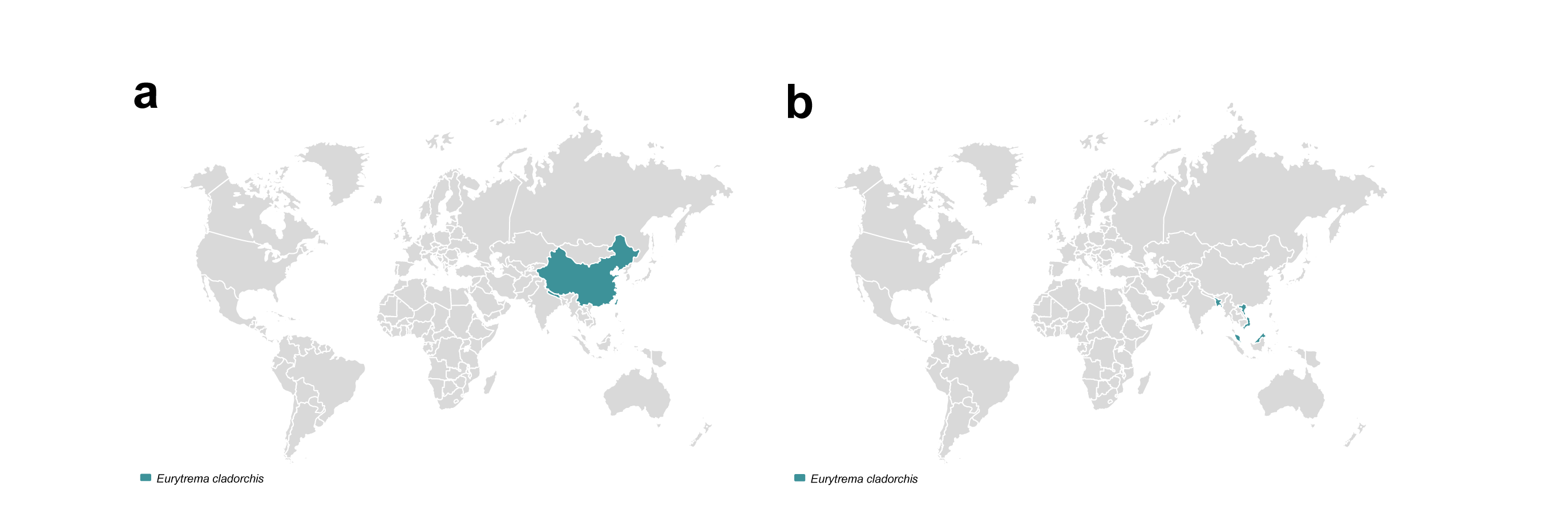

Supplement: Supplementary file 12 — Additional file 12: Fig. S10. Global distribution of Eurytrema cladorchis. (a) 1951–2000. (b) 2001–2023. [file 40249_2024_1208_MOESM12_ESM.tif]
